# Isotropic components of microseismic moment tensors at Utah FORGE reveal a diversity of fluid pathway creation processes in EGS development

**DOI:** 10.1038/s41598-026-42493-0

**Published:** 2026-03-10

**Authors:** Peter Niemz, Gesa Petersen, James Rutledge, Katherine Whidden, Kris Pankow

**Affiliations:** 1 University of Utah, Seismograph Stations, 15 South 1460 East, Room 211 FASB, Salt Lake City, Utah 84112 USA; 2 GFZ Helmholtz Centre for Geosciences, Telegrafenberg, Potsdam, 14473 Germany; 3 Santa Fe Seismic LLC, 4 Entrada Empinada, Santa Fe, 87506 NM USA; 4 Present Address: NORSAR, Gunnar Randers Vei 15, Kjeller, 2007 Norway

**Keywords:** Induced seismicity, Moment tensor, Utah FORGE, Isotropic components, Tensile shearing, EGS, Energy science and technology, Engineering, Solid Earth sciences

## Abstract

Full moment tensor (MT) inversion of induced microseismic events provides insight into stimulation processes during Enhanced Geothermal System (EGS) development. This study resolves the mechanisms of >180 microseismic events (local magnitudes 0.0-1.9) induced in two fracture zones during the 2024 stimulations at the Utah FORGE EGS test site. The remarkably homogeneous strike-slip mechanisms are generally consistent with the local stress field, but small rotations are observed between fracture zones of different stimulation stages. A significant proportion of events exhibit positive isotropic components, indicating simultaneous tensile opening. Within each fracture zone, the maximum isotropic component increases with injected volume. Interestingly, tensile opening associated with microseismic events is more prominent in fault reactivation than in zones where a hydraulic macrofracture is dominant. Non-double-couple MT components, particularly positive isotropic components, prove to be a powerful tool for characterizing reservoir development and differentiating between complex fracture networks and hydraulic macrofractures. These MT components may serve as an indicator of fault reactivation and a proxy for increased conductivity, highlighting the potential for improved reservoir characterization and management.

## Introduction

Anthropogenic interactions with the Earth’s crust, for example, caused by the exploitation of oil and gas, underground gas storage, mining, or geothermal energy production, alter the local subsurface stress field and inherently induce earthquakes. These earthquakes are often very small (microseismic events), but on some occasions, they are felt at the surface^1,2^. Due to the rising interest in exploiting deep geothermal energy in regions without a naturally permeable subsurface and an abundance of hot fluids, injection techniques adopted from hydraulic fracturing for hydrocarbon exploitation have been adapted for geothermal energy extraction. Geothermal temperatures required for energy production are often only reached at the depth of the basement rock. While the basement can be heavily fractured and faulted, it usually lacks conductivity due to a lack of connections between possible fluid flow paths. In enhanced geothermal systems (EGS), high-pressure fluid injections are used to break up the target rock, thereby enhancing conductivity in the geothermal reservoir and creating fluid pathways between the injection and production wells^3^.

The spatial patterns of induced microseismic activity are commonly used to track the development of the reservoir in general or the growth of hydraulic fractures in particular^4,5^.The microseismic activity is only one manifestation of the deformation taking place during high-pressure injections and only a small fraction of the deformation during EGS operations^6,7^. Compared to the impulsive seismic process of shear slip of rock in microseismic events, the purely tensile opening of a hydraulic fracture generates a slow and low-frequent signal^5,8^, that cannot be observed in the frequency band covered by microseismic monitoring setups. Consequently, the initiation and growth of a hydraulic fracture is an ‘aseismic processes’. A natural equivalent of hydraulic fractures and their aseismic behavior are magmatic dikes, which can propagate aseismically for many kilometers and may induce earthquakes in their vicinity^9,10^.

The detailed physical processes that trigger single, microseismic events during injections and their rupture process are still under debate, especially in EGS. For hydraulic fracturing, there are several models that explain the creation of fracture permeability^11^, the triggering of events^12^ and how these events contribute to the overall permeability of the reservoir. Processes dominating in sedimentary settings of hydrocarbon exploitation^5^ may be different compared to processes in the crystalline basement targeted in EGS^3^. From a few notorious examples, e.g., in Basel and Pohang^13,14^, it is well known that, in crystalline basement, preexisting faults play an important role for the largest seismic events induced during high-volume fluid injections.

The creation of fracture permeability in EGS was described before as a continuum between two end-members^11^: (1) pure tensile events build up a conductive fracture network by opening new hydraulic fractures, or (2) pure shear slip along preexisting small-scale fractures or zones of weakness that creates pathways by an offset of rough opposed fault surfaces^15^. Numerical models suggest that mixed-mode networks including the opening of hydraulic fractures and the activation of microseismic shear faults, are the most likely process for fracture permeability creation^16,17^ that constitutes the flow path in between wells.

**Fig. 1:**
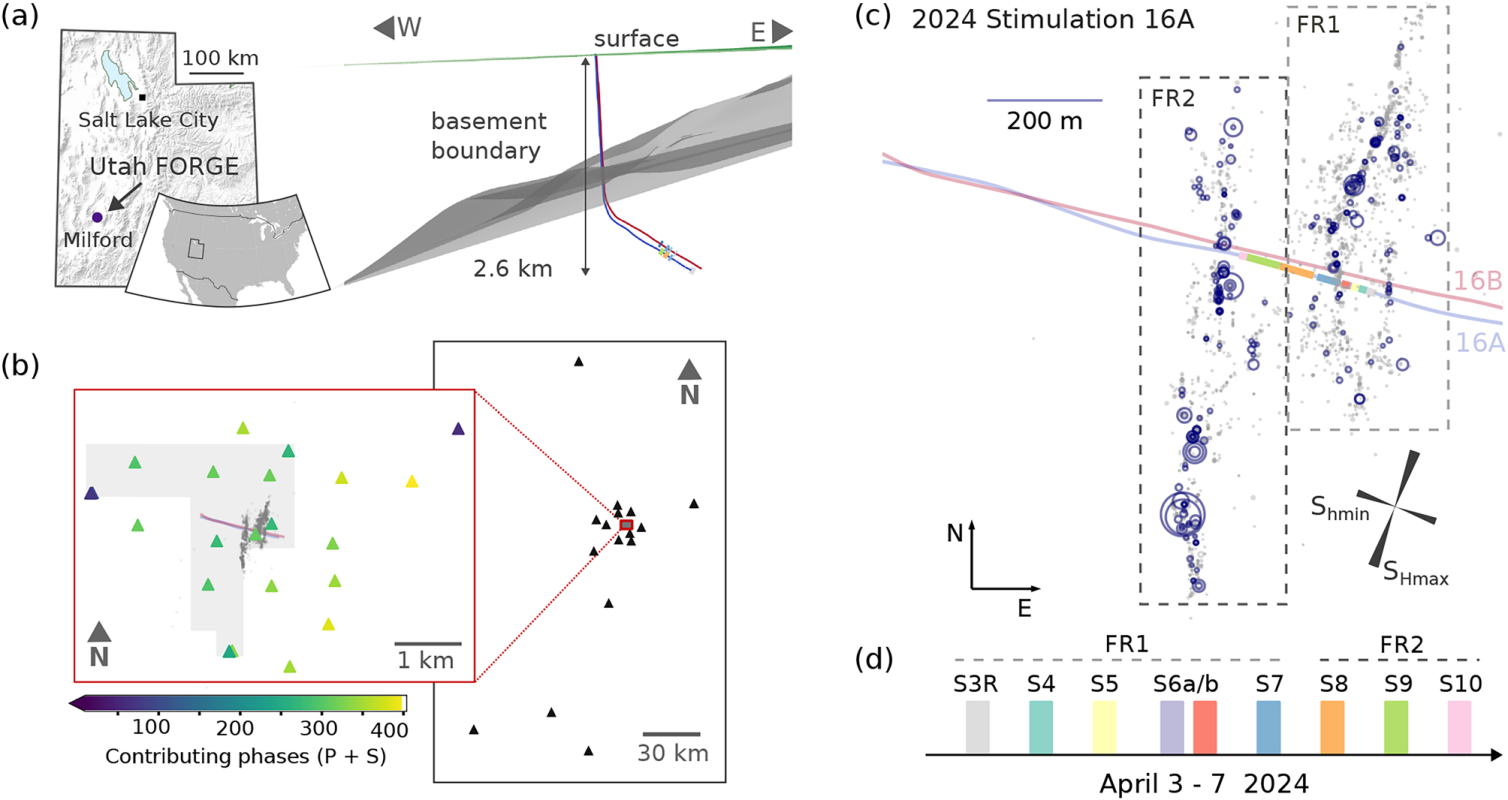
(**a**) Location of the Utah FORGE EGS site in central Utah (USA), and depth section showing the geometry of the injection (blue) and production borehole (red). (**b**) Map of stations used in the MT inversion. Permanent regional and local stations from the UU network and temporary nodal deployment at Utah FORGE during the stimulations in April 2024 (inset). Each triangle in the inset consists of 3x3 nodal geophone stations with a spacing of 20 m, color-coded by the number of contributing phases (P and S) in the moment tensor inversions. (**c**) Microseismic activity during the 2024 stimulations. There are two distinct microseismic features interpreted as two major fracture zones, FR1 and FR2^18^. Circle sizes are scaled by event magnitude. Larger blue circles represent target events for MT inversions (ML>0). (**d**) Timeline of stimulation stages with the main periods of activity of the two fracture zones and the corresponding stimulation intervals of the boreholes as marked in (**c**).

Due to the small number of field-scale multi-stage stimulation experiments in granitic rock, it is not clear if these models reflect the actual behavior of the rock in such a setting. In this study, we analyze source properties for EGS-induced earthquakes (local magnitudes ML<2) that accompanied the injection experiments and mapped the rupture growth during the 2024 stimulation at the Utah Frontier Observatory for Research in Geothermal Energy (FORGE) site^18^, a field-scale laboratory to develop and improve the technology needed for a safe and commercially viable application of EGS (Fig. 1).

By analyzing MTs of microseismic events induced during such a stimulation experiment at Utah FORGE, we add an observational perspective to the discussion of fracture permeability creation in EGS. Utah FORGE is an excellent test case to study the source processes of EGS-induced microseismic activity due to the dense seismic monitoring and comparably low-noise conditions. By design^19,20^, the injection pressure applied during the 2024 stimulation exceeded $Sh_{min}$, the least principal stress magnitude (Fig. S8). Hence, the initiation and further growth of hydraulic fractures, or more general tensile opening mechanisms, are possible and expected. During the stimulations at Utah FORGE, proppants, e.g. sands, were pumped down to hold fractures open at depth. This requires a volume expansion, implying the presence of hydraulic fractures.

The source processes of small to moderate earthquakes are commonly approximated as seismic point sources, mathematically described by the moment tensor (MT)^21,22^. Following the standard decomposition^21^, the MT is composed of an isotropic component (ISO), describing volumetric changes, a double-couple component (DC), describing shear slip on a fault plane, and a compensated linear vector dipole component (CLVD). The DC provides insights into active deformation on usually reactivated faults, which helps draw conclusions on (local) stress regimes^23,24^ and possibly unmapped faults.

Non-DC contributions (ISO and CLVD) are of utmost importance for our understanding of fluid-driven source processes during subsurface injections^25,26^. The ISO resolves symmetric volumetric changes, classically observed during explosions/implosions, e.g., in the context of the Comprehensive Nuclear-Test-Ban Treaty monitoring^27^. In EGS, the ISO may arise from the availability of additional fluid volumes within the source region, leading to an expansion within the geothermal reservoir. Together with a CLVD of same sign, the ISO can be interpreted as tensile faulting, the opening or closing of a fracture^28^. Non-DC components are often more difficult to resolve than the more dominant DCs^24,29^, being influenced by unmodeled path effects, anisotropy or complex rupturing, i.e., non-planar rupture surfaces or the overlap of multiple events close in time^29–32^. Because of these difficulties and the small magnitudes of microseismic activity resulting in reduced signal-to-noise ratio, a careful assessment of the resolution abilities and the robustness of results is required before any interpretation of the underlying processes that could explain ISO and/or CLVD (see *Methods* sections). When considering these limitations, MT inversion is a powerful tool for obtaining insights into the rupture process of earthquakes induced during EGS activity, as well as wastewater injections or gas extraction^25,26,33^.

To emphasize the difference between hydraulic fractures and microseismicity in this study, we use the term hydraulic macrofracture for a larger structure serving as a fluid pathway. It can either be a single large-scale hydraulic fracture or consist of a network of connected newly opened hydraulic fractures or dilated preexisting fractures. The opening and growth of a hydraulic fracture induces stresses at its tip, which may trigger microseismicity^34^, i.e., shear slip on microseismic faults. The slip on microseismic faults favorably-oriented in the regional stress field is also facilitated by fluids reaching a pre-existing fracture and reducing the effective normal stress on the fault plane^35^. In general, microseismic faults experience dominant shear slip, but may have isotropic (tensile opening) components. If tensile opening occurs during the first activation or later in the stimulation, these microseismic faults can contribute to the network of fluid pathway. Smaller new hydraulic fractures may form in between microseismic faults, but due to the slow tensile process, this is unlikely to be detected in seismic records.

We apply and adapt the state-of-the-art probabilistic full MT inversion tool *Grond*^36^ to study the complex interplay between faults and fractures at Utah FORGE. In the *Methods* sections we provide detailed information on the dataset and the waveform-based inversion procedure. Furthermore, we provide a comprehensive overview of quality control tests for our results, including the assessment of bootstrap-based uncertainties and synthetic-data-based resolution tests for the ISO. To facilitate and stimulate subsequent studies we provide the full set of results including tables of MT solutions but also detailed *html* inversion reports with figures of waveform misfits and uncertainties as an associated data publication.

The microseismic clouds of the induced seismicity observed during the stimulation and circulation experiments at Utah FORGE have been studied in detail to map the growth of the hydraulic macrofractures connecting the injection and production wells and the migration of fluids within the reservoir^18,37,38^. In this study, we follow up on these studies to gain insights into the rupture mechanisms of the microseismicity within the stimulated reservoir, aiming for an in-depth understanding of the events, the activated microseismic faults, and the differentiation of shear slip and tensile processes. We scrutinize those processes by first focusing on the results with respect to the DC solutions and the ISO, before discussing their physical implications with respect to preexisting structures, stress field, injected volume, and hydraulic fracture growth. The inclusion of non-DC components helps differentiate a wide range of processes, down to the scale of single microseismic events driven by hydraulic fracture development and fluid-forced fault reactivation.

## Mechanisms of microseismic activity at Utah FORGE 2024 stimulations

**Fig. 2:**
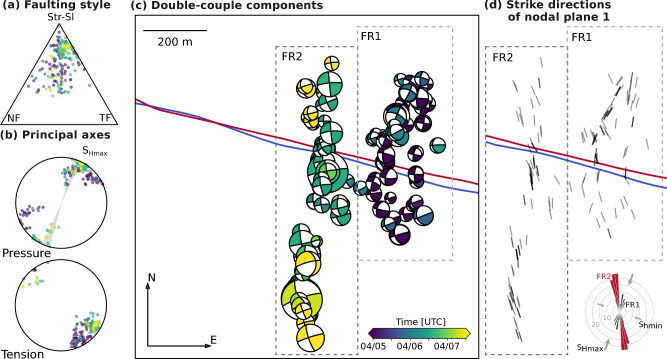
Overview of double-couple MT components color-coded by event origin time. (**a**) Triangular source-type diagram for 189 MT solutions. (**b**) P- and T-axes of the mechanisms with the local maximum horizontal stress direction $S_{Hmax}$ between N15$^\circ$E and N25$^\circ$E^39^ for comparison. (**c**) Map-view of the DC components. Blue and red lines represent the injection and production boreholes (Fig. 1). (**d**) Strike direction of the approximately north-south-oriented first nodal planes to emphasize the orientation of the DCs shown in (**c**). The inset in the lower right corner shows the frequency of strike directions of the first nodal plane for FR1 (black) and FR2 (red) in 5-degree bins, along with the local stress field for comparison. To overcome the 180$^\circ$ ambiguity of the strike direction due to steep dipping faults we include both strike and strike+180$^\circ$ resulting in twice the number of strike directions compared to the event number.

We derived MT solutions for 189 out of 229 microseismic candidate events induced during the 2024 stimulations at Utah FORGE with moment magnitudes (Mw) from 0.6 to 2.3 (Fig. 2, see Supplementary Fig. S1 for a comparison of local and moment magnitudes, and *html*-reports in data publication for detailed information on each event’s resolution). This subset of events covers the entire seismogenic volume active during the stimulations and accounts for about 90% of the total seismic moment release. The events are located in two main fracture zones activated during the 2024 stimulations^18^. The first fracture zone (FR1), extends the previously active zone from the 2022 stimulations^37^ around stage S3R outwards and remains active until stage S7 with lingering seismicity also in later stages. The second fracture zone (FR2) is active in stages S8 to S10, showing a clear spatial offset between the injection intervals and the microseismic activity (Fig. 1 c and d).

The majority of MTs have a strike-slip mechanism with nodal planes oriented NNW-SSE to NNE-SSW and ENE-WSW to ESE-WNW. The NNW-SSE to NNE-SSW striking nodal planes are in agreement with the elongated distribution of the microseismicity (microseismic cloud) of the fracture zones FR1 and FR2^18^ (Fig. 1c and 2). While strike-slip mechanisms are dominant, a few events show some obliqueness tending towards normal faulting (triangle diagram in Fig. 2a). The pressure (P-)axis and tension (T-)axis of the mechanisms are sub-horizontal and oriented in NNE-SSW to NE-SW and SE-NW to SSE-NNW direction, respectively (Fig. 2b, Supplementary Fig. S2). The mechanisms of the later-occurring events in the western fracture zone FR2 (green to yellow colors) show a small rotation of the nodal planes and P- and T-axis (approximately 30$^\circ$; Fig. 2b and c) compared to the mechanisms of the earlier events in FR1 (blue colors).

**Fig. 3:**
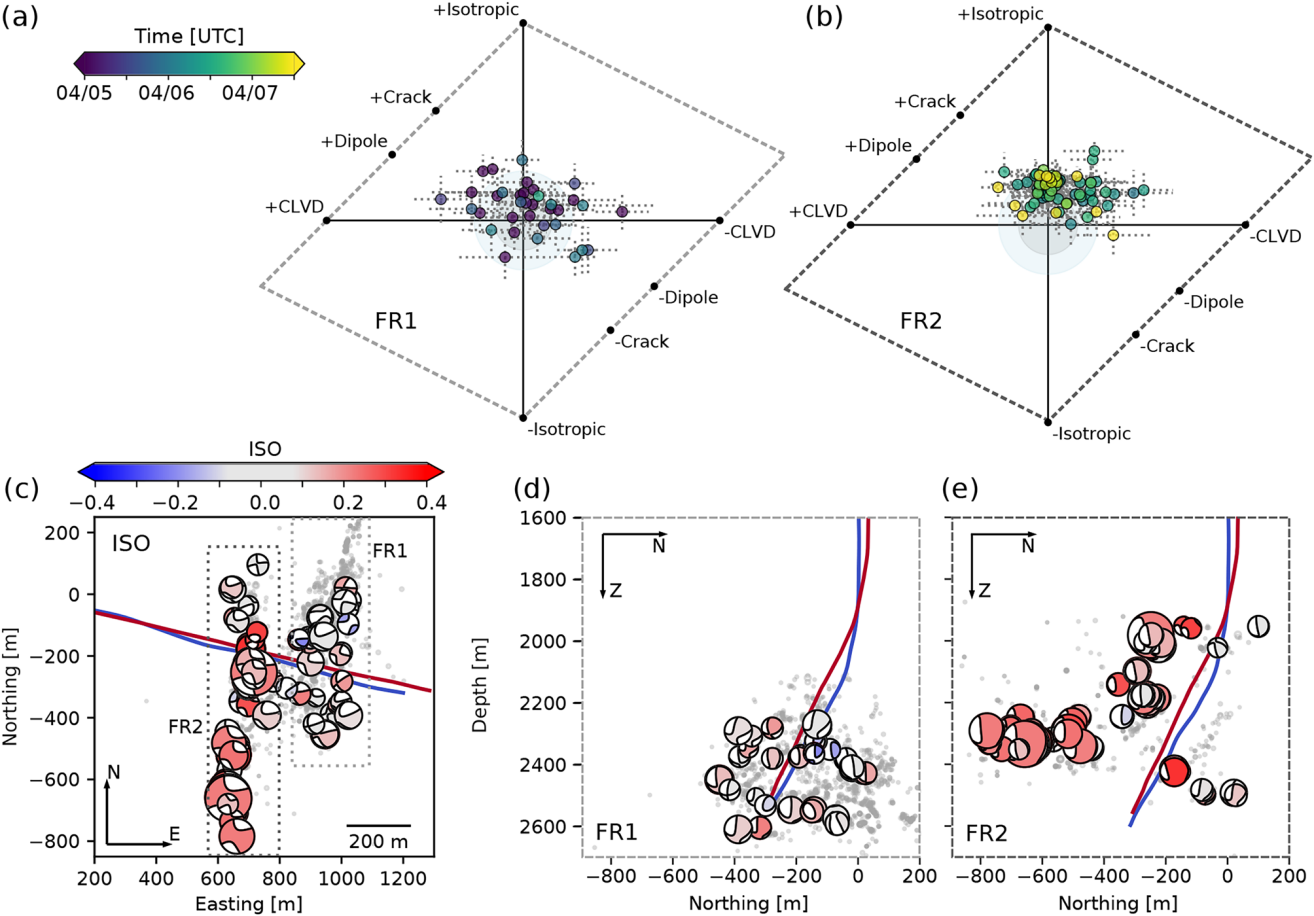
Non-DC components of the MT solutions. (**a**) and (**b**) show the Hudson plot representation of non-DC components with standard deviations (grey dashed lines) for FR1 and FR2, color-coded by the event origin time as in Fig. 2. Circles in the background mark non-DC contributions of 15 % and 25 %. (**c**-**e**) show the best fitting full MT solutions for 109 well-resolved events color-coded by the signed isotropic contribution in map view (**c**), and in two depth sections (**d**) and (**e**), one for each of fracture zones, FR1 and FR2.

It is important to state that for all analyzed events, the majority of the seismic moment is released by shear slip, which is described by the DCs (Supplementary Fig. S3), ranging between nearly 100% to a minimum of about 60% of the total moment release. After the manual quality control of the MT solutions and the application of the ISO uncertainty threshold (see *Methods* sections), we retain stable isotropic components for 109 events showing predominantly positive ISOs (Fig. 3a,b). The CLVDs are in general less well resolved, showing positive and negative signs with larger standard deviations in their bootstrap-based result distributions (Fig. 3a,b and Supplementary Fig. S6).

**Fig. 4:**
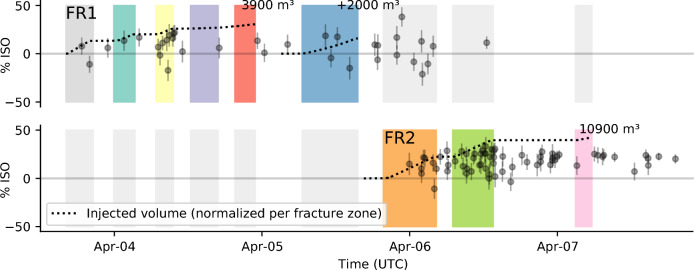
Isotropic MT contribution and injected volume for fracture zone FR1 and FR2. Error bars show the standard deviation of the isotropic contribution obtained from bootstrapping. The injected volume (dotted lines) is normalized per fracture zone. Stage S7 is normalized on its own as it formed a spatially distinct sub-feature within fracture zone FR1^38^. Only events with an ISO standard deviation below 12% are shown. Background colors indicate the injection intervals as introduced in Fig. 1. See supplement Fig. S5 for a version including all ISOs.

Fracture zones FR1 and FR2 show distinct differences with respect to the ISO (Fig. 3a,b). The events induced in the later stages of FR2 include larger, predominantly positive ISOs, many of which exceed 20%. The events in FR1 have smaller positive ISOs. In FR1, four events have negative ISOs exceeding −15%, in FR2 no event has a significantly large negative ISO. Fracture complexity and pressure release (see FR1 after the stimulation of FR2 starts, Fig. 4) might lead to closing processes that are reflected in negative ISO. However, as larger negative ISOs are rare in our data set, we refrain from any further interpretation.

## The physical interpretation of the DC

The basement at Utah FORGE was formed between multiple stages of magmatic activity as well as extensional Basin-and-Range tectonics. Thus, we can expect a complex natural fracture network with slightly variable fault orientations^40^. The low-magnitude natural seismicity in the wider region shows normal and strike-slip faulting^41,42^. Local stress estimates from drilling-induced fractures and image logs in the injection well and the production well constrain the orientation of the maximum horizontal stress $S_{Hmax}$ to N15$^\circ$E to N25$^\circ$E at reservoir depth^39^, similar to the regional stress field ($\sim$N30$^\circ$E)^43^. Under purely tectonic stresses, preexisting faults in a strike-slip regime are most likely to slip when they are oriented at an angle of approximately 25–30$^\circ$ to $S_{Hmax}$^44^.

Throughout this study, we interpret the first set of nodal planes with the NNW-SSE to NNE-SSW orientations as the causative fault planes for three reasons: Firstly, these nodal planes align with the microseismic cloud orientation. Secondly, nearby geological features, such as mountain ranges and their bounding faults, follow an approximate N-S trend. Thirdly, the positive ISOs, which we interpret as a proxy for a tensile earthquake (see details in next subsection), indicate an opening process during the rupture. Such an opening would have to occur against the $S_{Hmax}$ direction if the secondary set of nodal planes is the causative one, which is highly unlikely.

The strike of the microseismic cloud of the first fracture zone FR1 follows the regional $S_{Hmax}$, as observed in previous phases of the Utah FORGE^38^ and in other EGS-related experiments^13,34,45^. The strike of the microseismic cloud of FR2 is rotated, striking N to NNW^18^. This rotation between FR1 and FR2 is also seen in the P and T axes of the corresponding MTs (Fig. 1c, 2b, and Suppl. Fig. S2) and the strike directions of the fault planes of the dominating strike-slip events (Fig. 2d).

The microseismic fault planes resolved by the moment tensors of FR2 are perfectly oriented for slip relative to $S_{Hmax}$, as can be seen by the excellent agreement of the P-axes of events in FR2 and $S_{Hmax}$ in Fig. 2b. The almost perfect orientation for slip in the regional stress regime for FR2 and the extremely high internal similarity of P- and T-axis orientations among events in the southern branch and in the central branch of FR2, as well as the alignment of events in the microseismic cloud (Fig. 2), may indicate that the stimulation activated preexisting faults that ruptured stepwise as fluid entering the fracture zone FR2 reduced the effective normal stress. Such shear slip on optimally oriented faults is expected to extend further from the treatment well than tensile crack growth due to the lower threshold pressures required to induce shear slip.

Contrary to FR2, the P-axes and the strike directions of the DCs of FR1, especially in its northern branch, indicate fault orientations less favorable for slip because the NNE-SSW fault planes align close to $S_{Hmax}$ (Fig. 2d). A localized stress perturbation imposed by the tip of a hydraulic macrofracture growing in the direction of $S_{Hmax}$ can explain the observed microseismic shear events in FR1, even when the shearing planes are not prone to slip in the regional stress field.

The previous stimulation of the reservoir in fracture zone FR1 during the 2022 stimulations may contribute to the increased heterogeneity of fault strikes compared to FR2, including a possible shear reactivation of previously opened macrofractures besides the initiation and growth of new hydraulic macrofractures. South of the two wells, the mechanisms of FR1 show a similar orientation as in FR2, suggesting similar processes.

We observed a large spatial offset between the injection intervals S8-S10 and the onset of microseismicity in FR2. As the microseismicity is clearly triggered by the injection, the offset suggests an aseismic propagation along a preexisting conductive feature (e.g. a preexisting fracture or drilling induced damages in the vicinity of the well) before the onset of microseismicity on a pre-existing fault. This suggested conductive feature is not mapped by microseismic activity since it strikes sub-parallel to the well doublet, which is not optimal for shear slip. Eventually, the fluid reached a fault (zone) prone to slip^18^ and produced detectable microseismic activity. Hence, the MTs illuminate only those microseismic faults prone to slip.

## The physical interpretation of the decomposed full moment tensor

The first insights into hydro-mechanic processes based on the DCs are complemented by analyzing the non-DC contributions. While the decomposition of the MT into an ISO and a deviatoric component is unique, the subsequent decomposition of the deviatoric part is not unique. Following the standard decomposition, as done in this study, the deviatoric part of the MT is further decomposed into a DC and a CLVD.

The contribution of the DC to the full MT ranges from 60% to nearly 100%, indicating a dominance of shearing. Nonetheless, the non-DC components are significant for many events in this study, and the evolution of non-DC contributions over time bears the potential to obtain more insights into reservoir dynamics. Using the standard MT decomposition and the bootstrap-based uncertainties, we conclude that we can well resolve the DC and, for approximately 60% of the events, also the ISO, while it remains challenging to resolve sufficiently stable CLVDs (Supplementary Fig. S6). An alternative MT decomposition approach splits the deviatoric MT into a major DC and a minor DC^21^, reflecting faulting complexity or the overlap of two coinciding independent events, as suggested, e.g., to explain CLVD components of wastewater-injection-induced microseismic events^25^. For our case, for both types of decompositions (standard and alternative), the (major) DCs, constituting the main contribution of the deviatoric MTs, are well recovered, and the remainder CLVD or minor DC reflects the same instabilities. The resulting minor DCs describe normal faulting with strike direction flipping by 90 degrees, equivalent to negative or positive CLVDs in the standard decomposition.

Contrary to the CLVD, we obtain well-resolved, significant positive ISOs for many events, especially in fracture zone FR2, indicating a volumetric expansion. Our MT inversions show that the maximum isotropic contribution to the full MT increases with the injected volume in both main fracture zones, while at the same time microseismic events with smaller isotropic components are still observed (Fig. 4). The maximum ISO eventually reaches upper bounds of 25–40%, reflecting the overall dominance of shearing (DC) in the studied MTs. A pure tensile earthquake representing the opening of a fracture, here under fluid-forcing, would be described by a positive ISO and a positive CLVD^28^. Due to the poor resolution of the CLVD, we have to limit our interpretation to the ISO. A radially symmetric expansion, as represented by the ISO without CLVD, can be observed for underground explosions but is not expected during high-pressure injections into a pre-fractured medium under stress. Therefore, we use the ISO as a proxy for the gradual change from shearing to tensile earthquakes, even if we do not resolve a positive CLVD.

In literature on induced seismicity, the relation between injected volume and maximum magnitude is well-established^46,47^. The increase of maximum ISOs of the microseismic events with increased injection volume, as observed here, has not been commonly reported or studied. Generally speaking, an increase of the maximum ISO over time may reflect changes in the medium, in the rupture process, or in both. Seismic anisotropy in the source medium is expected to be mostly mapped into the CLVD, but not (or to a very small amount) into the ISO^31,48^. We cannot completely rule out a minor influence of unmodeled, gradually increasing anisotropy at the source. However, the fact that fracture zone FR1, which had been stimulated in previous experiments, does not show large ISOs indicates that source media anisotropy caused by the stimulation itself is unlikely to be the cause for the observed volumetric components.

The more pressurized fluids are pumped into the reservoir, the more fluids potentially reach microseismic faults, which can then be forced to open while slipping. Accordingly, the maximum opening at a point in time is determined by the increasing amount of available fluid. Therefore, we attribute the observation of an increasing maximum isotropic component with increased fluid supply to the increasing contribution of the microseismic faults to the network of fluid pathways. While opening components can become more important in some parts of the reservoir, microseismic faults that are in stress shadows impeding opening^49^ or microseismic faults that are not interlinked, thus having limited fluid supply, can still slip under pure shear without any ISO contribution.

## Interaction of fluid injection and microseismic shear events with tensile opening

The conductivity of the reservoir after the 2024 stimulation was larger compared to the 2022 stimulations, which was attributed to the usage of proppants that keep hydraulic fractures open^50^. While we cannot separate the contributions of each fracture zone to the overall reservoir conductivity enhancement, we observe spatial differences in the microseismic footprint that suggest different driving processes.

**Fig. 5:**
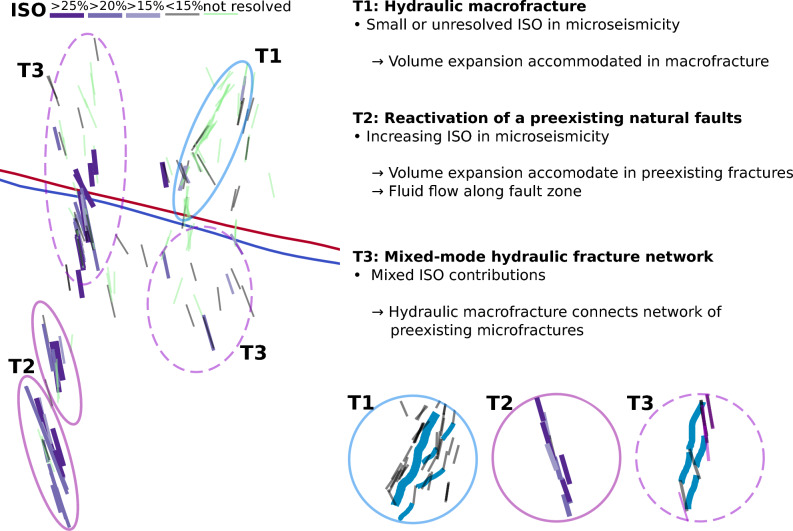
Conceptual representation of the three different fracturing types proposed for the 2024 stimulations. The left panel shows the causative nodal planes of the MTs, color-coded by the amount of isotropic opening. The inset sketches in the circles represent the conceptual fracture types (not to scale). Gray to purple lines indicate fault planes with color-coded isotropic contributions (see legend in a), blue lines represent hydraulic fractures. Fault plane length scaled by seismic moment assuming a constant stress drop of 1MPa^51^.

Based on the joint observations of (a) DC strike directions of the microseismicity, being a proxy for the preexisting fracture network, (b) the migration direction within the microseismic cloud, mapping the growth of a hydraulic macrofracture, and (c) the contribution of the ISO as a simplified metric describing the opening during the microseismic rupture process, we suggest the presence of different types of hydraulic processes acting during the 2024 stimulations at Utah FORGE (Fig. 5).

**Type (T1) - Dominant hydraulic macrofracture:** In the northern part of FR1 (Fig. 5), the elongation of the microseismic cloud in the $S_{Hmax}$ direction (see also Fig. 1) and the almost perfect alignment of MT fault planes with $S_{Hmax}$ suggest the presence of a hydraulic macrofracture, because the microseismic faults are less favorable for slip in the undisturbed regional stress field. Close to the wells in facture zone FR1, the rock was already stimulated in 2022 with further hydraulic macrofracture growth during the 2023 circulations^38^. Therefore, fracture reopening and further growth are expected during the 2024 stimulation, with injection pressures exceeding $S_{hmin}$. The hydraulic macrofracture grows mainly aseismically but is mapped by the activation of microfractures^5^ ahead of the hydraulic fracture tip. In this domain, the ISOs are either smaller or not resolved (see also Supplementary Fig. S7), indicating that the microseismic events do not play a major role in the initial creation of fluid pathways. This first interpretation of type T1 tends towards one endmember of fracture permeability creation^11^ with an important role of a hydraulic macrofracture. In addition to stress changes at the hydraulic fracture tip, hydraulic connections may develop between the hydraulic macrofracture and microseismic faults in its vicinity. Fluids reaching microseismic faults may reduce the effective stresses and microseismic faults may slip despite their comparatively unfavorable fault plane orientation with respect to the regional stress field.

**Type (T2) - Reactivation of a preexisting natural fault (zone):** In the southernmost part of FR2, we resolve tightly clustered, well aligned microseismic faults with consistently large ISOs (Fig. 5). The tight alignment of the microseismic events in the strike direction of the MT fault planes indicates the presence of an elongated, preexisting fault zone. The presence of preexisting faults explains the rotation of the microseismic cloud with respect to the regional stress field as well as compared to fracture zone FR1. In such a setting, it is energetically more favorable to activate the preexisting fault(s) instead of opening a new hydraulic fracture. The observation of the isotropic component of the microseismic events growing in time supports the interpretation that a preexisting highly conductive fault zone is activated via microseismic events and constitutes a significant reservoir flow path. In this case, also shear dilatation^52^, the offset of opposed rough fault surfaces, may play a more important role in the increase of permeability within the reservoir^53^. The increased role of the microseismic events in the fracture permeability creation tends towards the second endmember, the dominance of hydroshearing^11^.

**Type (T3) - Mixed-mode hydraulic fracture network:** In the central and northern parts of FR2 and in the southernmost part of FR1 (Fig. 5), we observe an elongation of the microseismic cloud in the N-S direction, while the MT fault planes strike in NNW-SSE direction. The MT fault planes indicate a dense network of preexisting microseismic faults, which are well oriented for slip. However, the sub-parallel geometry of the microseismic faults provides little connectivity. To establish connectivity for fluid flow, a mixed-mode hydraulic fracture network is needed that experiences both shear slip reactivation with tensile opening components and the aseismic opening of new hydraulic fractures. In this network, the hydraulic fractures create connections between the microfaults. This is similar to a fracture mesh model suggested for volcanic intrusions^54,55^ or hydraulic fracturing in sandstone and shale^56,57^ and corresponds to the mixed-mode stimulation numerically modeled for EGS^16,17^. The differential slip of neighboring en-echelon faults can create extensional cavities that further facilitate the opening of hydraulic fractures^58^. The availability of pressurized fluids at a preexisting microseismic fault depends on the connection to the reservoir and influences the amount of opening, thus the ISO. Also for this type, local stress perturbations (e.g., at the tip of a hydraulic macrofracture) or the reduction of the effective normal stress due to very small amounts of fluid can still cause pure shear slip on preexisting microseismic faults prone to slip, leading to a mixture of pure shear and partly tensile microseismic events.

In all three types, the preexisting fracture network plays an important role and small rotations in those fracture sets lead to different roles of microseismic events regarding the fracture permeability creation. In T2, the microseismic events directly reflect the initial activation of a preexisting conductive fault zone, which is expected to dominate the creation of fracture permeability. For T1 and T3, microseismicity reflects a more granular process of fracture permeability creation, building a mesh of newly formed hydraulic fractures and preexisting fractures–activated by shear slip as microseismic events or opened aseismically as hydraulic fractures. The hydraulic macrofracture described for T1 is most probably not a single hydraulic fracture but a very dense network of fractures closely aligned with the direction of $S_{Hmax}$, constituting a fracture mesh similar to T3, but the mesh develops in a much narrower zone due to the parallel orientation of $S_{Hmax}$ and the strike of the fracture set.

## Discussion and conclusions

We provided a quality-controlled analysis of MTs from microseismic events induced during the 2024 stimulation experiments at Utah FORGE. The strike-slip faulting mechanisms of the events show a remarkable homogeneity. For the non-DC components, we limit our interpretation to the isotropic components which we use as a proxy for tensile opening mechanisms. In a joint interpretation of the fault plane orientations, the strike of the microseismic cloud, and the well-resolved ISO components we describe a remarkable diversity comprising three hydraulic reservoir enhancement types only separated by a few hundred meters. All three types underline the importance of the geometry of preexisting fractures and their connectivity within the fractured basement rock. Unconnected preexisting natural fractures can be efficiently connected by new, induced hydraulic fractures. On the flip side, a network of fractures or faults confined to a narrow zone and interlinked might, in the worst case, lead to the activation of larger fault zones^14^ as it can transport fluid quickly beyond the targeted reservoir, increasing the risk of reaching larger faults prone to slip. In the best case, the preexisting fractures contribute to an efficient and safe conductive network. Analyzing and monitoring the link between hydraulic fracturing and induced microseismicity, as done in this study, is a requisite for safe reservoir development. We observe an increase of the maximum ISO component of the inverted microseismic MTs with injected volume during the stimulation at Utah FORGE. We attribute this increase to a more important role of the microseismic fault network in generating fluid pathways, thus indicating a high complexity of the fracture networks. In those networks, preexisting microseismic faults can accommodate parts of the volume increase in shear-tensile mechanisms. Consequently, fluids can travel along newly opened hydraulic fractures and natural, preexisting conductive features^59^.

This leads to a paradox observation: The microseismic events mapping the reactivation of a preexisting fault zone optimally oriented for shearing show larger positive ISOs, whereas the observed microseismic events that closely align and map the hydraulic fracture growth show little tensile opening.

While resolving ISOs (and potentially CLVDs) requires a thorough, challenging analysis of resolution and uncertainties, the benefits for characterizing the reservoir development are significant. The non-DC components potentially provide a robust way to differentiate between complex fracture networks and single, isolated hydraulic macrofractures. For future work we suggest to asses the relation between increased ISOs of the microseismic events and the reactivation of preexisting natural faults across a variety of fluid-injection scenarios. If a generalization is found, the presence of significant ISOs along fracture zones favorably oriented for slip can reveal if distal microseismic events are a response to a pressure connection originating from the treatment well or, if instead, they represent significant fluid flow paths - ultimately improving our ability to predict and manage subsurface fluid-fault interactions.

## Methods

### Dataset of the April 2024 stimulations at Utah Forge

Utah FORGE is composed of two deep, deviated wells with a maximum depth of $\sim$2600 m (injector 16A(78)−32 and producer 16B(78)−32) and four monitoring wells with depths of 998 to 2288 m. The injection and production wells are vertical down to $\sim$1800 m below the surface. Below this depth, they are deviated at $\sim$65$^\circ$ for a distance of 1500 m (Fig. 1a). In the deviated section, well 16B is located approximately 100 m above well 16A^60^.

The field scale laboratory is monitored by a dense local seismic network (Fig. 1b), as part of the regional seismic network (network code UU) operated by the University of Utah Seismograph Stations^61^. The locally densified network comprises seven broadband sensors at the surface (station codes FORx) and six sensors in shallow boreholes at depths ranging from 26 to 41 m (station codes FSBx). The seismometers are installed in rings with radii of 3 and 8 km around the injection site. The closest station to the injection (FORK) is installed in a 300 m deep borehole^62,63^.

This study focuses on the 2024 stimulations of well 16 A from April 3 to 7. Previous activities at Utah FORGE include the initial stimulations in 2022 and a circulation test in 2023^38,63^. The 2024 stimulations include a refracturing stage at the toe (S3R in Fig. 1d, reactivating stage S3 from the 2022 stimulations) and seven additional fracturing stages moving toward the heel of the injection well (stages S4-S10). The injected volume per stage ranges from approximately 500 m$^3$ to 5,600 m$^3$.

For the April 2024 stimulations at Utah FORGE, we deployed an array of 16 nodal geophone patches, complementing the permanent network stations to achieve the azimuthal coverage required for MT inversions and precise locations (inset in Fig. 1b). Each patch consists of 9 equally-spaced geophones in a small square grid with a side length of 20 m and an instrument spacing of 10 m^18^. In our MT inversions, we use the direct stack of the raw waveforms of each 3x3 mini-array to take advantage of enhanced SNRs^18^. The geophones were set up with hand-held magnetic compasses; therefore, we carefully assessed the orientations of the horizontal components prior to inverting for the source mechanisms (Supplementary paragraph S2.2 and Fig. S9) to rule out systematic offsets that could bias the stacked waveforms from the geophone patches.

### Probabilistic MT inversion

We use the probabilistic inversion tool *Grond*^36,64,65^ to target the 229 candidate events with local magnitudes ML 0.0 to ML 1.9. The probabilistic MT inversion allows the study of rupture processes of tiny earthquakes, including a comprehensive assessment of uncertainties, resolution limits, and trade-offs of inversion parameters. Each inversion is performed in 100 bootstrap chains with variable random weights of the input data in addition to one global chain. In the global chain, all station-component based input data are weighted equally after applying a station-distance correction to compensate for the fact that stations at further distances have smaller amplitudes and thus would contribute with a smaller misfit^66^. By applying random weights to the station-component based inputs in 100 different bootstrap chains, we assure that single stations do not bias the results, e.g. when the velocity model is less appropriate for particular stations. The ten best-fitting models of each chain (1010 models in total) are used to assess the solution’s stability.

We simultaneously invert for seven parameters: the six independent MT components and the duration of the source time function, which is approximated as a half-sinusoidal. For each inverted parameter *Grond* provides probability density function plots, allowing for an assessment of uncertainties and parameter trade-offs based on the bootstrap chains (see Data Availability for access to the *html*-reports for each inverted event).

To forward model the synthetic waveforms for the inversion, we use precalculated Green’s Function (GF) databases computed using the qseis code for layered viscoelastic half-space earth models implemented in fomosto^67,68^. The local two-layer velocity model^69^ has a first layer (0–1.69km) with $v_{p}$=3.4 km/s and $v_{s}$=1.95 km/s and a second layer (1.69–17.25 km) with $v_{p}$=5.8 km/s and $v_{s}$=3.4 km/s. For computational efficiency, we increase the grid spacing of the GF databases with increasing epicentral distance. For most events, we incorporate stations from 0.5 km to 40 km distance. For the largest events we use stations to 150 km. For epicentral distances to 5 km we use a grid spacing of 50 m in depth and distance; to 40 km, the grid spacing is set to 100 m in depth and 200 m in distance; and to 150 km, the grid spacing is 250 m in depth and 500 m in distance. All databases were calculated at a sample rate of 50 Hz with source depths reaching from 500 m to 4500 m.

Using stations at different distances, whenever possible, is important to cover a larger portion of the focal sphere as the phases arrive at more distant stations with a different incidence angle. In addition, more distant stations are less affected by the dipping bedrock boundary (Fig. 1). Thus, a regional Green’s function database is well suited.

The 2-layer velocity model is a strong simplification of the 3-D structure with a dipping bedrock layer. Unfortunately, at the moment of writing this paper, no 3-D waveform modeling tool exists that is sufficiently fast to forward model tens of thousands of tested models for the inversion of even a single event mechanism. Additionally, we use precise manual arrival picks of P- and S-phases to define the time windows, thereby removing any travel-time dependence. The optimization algorithm is a two-stage method with an initial uniform search stage and a second directed search stage, in which new models are drawn based on previous low-misfit models. The algorithm is described in detail on *Grond’s* webpage: https://pyrocko.org/grond and in^64,65^.

The inversion tool *Grond* allows for a flexible combination of different input data types in the MT inversion^24,36^. We use combinations of time domain waveforms, cross-correlation-based waveform misfits, and frequency domain amplitude spectra as an input for the inversion, as well as polarities with a much smaller weighting (0.1, vs. 1.0 for the two other input types). We use P phases recorded on the vertical components and SH phases on the transverse components of the seismograms, but exclude the radial components, as they appear to be noisier and are easily influenced by P coda, P-to-S conversions, or inadequacies in the velocity model. Misfits are weighted group-wise so that misfits of the P-wave cross-correlations have the same influence on the results as misfits of the S-wave cross-correlations, the P-wave spectra and the S-wave spectra. Using spectra and cross-correlations reduces the dependency on the 1-D velocity model and thus stabilizes the inversion compared to using the time-domain waveform fitting alone^24^. We bandpass filter the waveforms between 3 and 7 Hz, and use a time window length of 0.34 s - 0.4 s around the manually picked P and S phase arrival times. The small epicentral distances and the dipping boundary between the basin sediments and the granitoid basement lead to complexities in the full waveforms, e.g., from high-amplitude converted PS phases originating from the basement boundary. We minimize the bias resulting from using a 2-layer model by fixing the event hypocenters, which were relocated within a 3-D velocity model^18^. The separate inversion of short time windows containing only P phases or S phases avoids mismatched converted phases that can bias the inversion. To further avoid mismatching phases, we align the manual phase arrival picks with the theoretical arrivals to focus the inversion on fitting the waveform features. The down-weighted polarities mainly help to assess the sanity of the resolved focal mechanism by comparing whether the waveform-based derived MT fits the polarity readings^70^.

### Quality control, uncertainties and resolution tests of MT inversion results

The resolution of seismic MTs strongly depends on the available dataset, including the availability of well-distributed seismic stations, the waveform quality/signal-to-noise ratios, and a sufficient knowledge of the velocity model representative of the travel path. Additionally, if no centroid inversion is performed simultaneously, the centroid location, including its depth, must be known accurately. All of these factors can influence the quality of MT solutions, which should not be provided without estimates of uncertainties and a careful evaluation of the resolution limits. In this study, we relied on a careful evaluation of bootstrap-based uncertainties combined with synthetic waveform-based resolution tests to ensure the quality of our results.

#### Uncertainties from bootstrapping and constraints from picked polarities

We use the ensemble of the ten best results of each of the 101 bootstrap chains to assess uncertainties and trade-offs of the inversion parameters. From the ensemble of each event, we obtain a mean MT and its standard deviations, as well as the overall best-fitting MT solution. Analysis plots for one well-resolved and one insufficiently resolved example event are provided in the supplement (Supplementary Fig. S10 and S11). Further, the best MT, the mean MT and the standard deviations of all events are provided in csv tables. The tables and the detailed html inversion reports including the described figures are provided as a data publication.

We manually inspected the solution quality for each event. This includes a visual check of waveform and spectral fits, as well as a visual comparison of the best and mean MT solutions. A first-order assessment of the ensemble stability is achieved through the *fuzzy* beachball representation, an overlay of all ensemble solutions (Supplementary Fig. S10). A stable solution shows sharp separations between compressional and extensional regions, while an unstable solution remains fuzzy. Additionally, we check the consistency between manually picked polarities and the radiation pattern in the fuzzy beachball representation.

Next, we quantitatively compare the best and mean solutions. Stable solutions show similar contributions of the standard decomposition into DC, CLVD, and ISOs (Supplementary Fig. S10; see also Supplementary Fig. S3 for a histogram of all decompositions and S6 for the standard deviations of ISO and CLVD). For the DC stability, we consider the smallest rotation between the DC of the best and the mean MT (Kagan angle, see also Supplementary Fig. S12) as well as the distribution of strike, dip, and rake values from the entire ensemble.

The contributions of non-DC components are evaluated via the Hudson plot^71^, which depicts the ensemble solutions within a coordinate system of signed ISO and CVLD. A tight scatter cloud within the Hudson plot points towards a stable resolution of the non-DC components. Furthermore, we can readily identify classic trade-offs between ISOs and CLVDs represented by an elongated distribution between negative and positive crack solutions. If such a trade-off or other limitations of the non-DC resolution are found, we exclude the non-DC components but still inspect the DC, which can nevertheless be well-resolved. In such a case, we only use and report the DC in our interpretations.

In general, events with smaller magnitudes have lower SNRs, which may potentially bias the MT solutions towards larger non-DC components, accommodating unmodeled noise signals within the waveforms. Therefore, we tested for this apparent dependency of the non-DC contribution on the event magnitude by plotting the CLVDs and ISOs over magnitude (Supplementary Fig. S6). We found no trend of larger non-DC components with decreasing magnitude and concluded that the resolved non-DC components are independent of the noise level for our quality-controlled full MTs. Similarly, we tested for the potential depth dependence of the ISO and CLVD (Supplementary Fig. S6), which shows no dependency, ruling out a first-order influence of the simplified velocity model on the MT solutions.

#### Synthetic waveform-based resolution tests of non-DC components using synthetic data

**Fig. 6:**
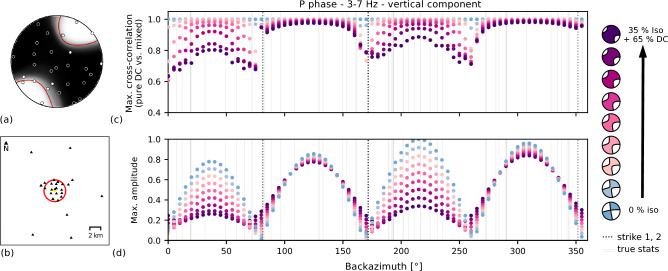
Resolution test for isotropic components based on synthetic data for a representative strike-slip event (here shown for the P phase on the vertical component). (**a**) Fuzzy moment tensor showing the inversion result for the example event with an isotropic component of 30 %. (**b**) Test network geometry (red circle) and actual station distribution (black triangles). The circle consists of 72 virtual receiver locations at 2 km epicentral distance to the source. (**c**) and (**d**) show results of the comparison of synthetic waveforms obtained from a pure DC (blue) and mixed-mechanisms generated from the same DC and a positive isotropic component varying from 5 % to 35 % (see color scale on the right). (**c**) Maximum cross-correlation values of waveforms forward modeled using a pure DC and the mixed (DC+ISO) sources. (**d**) Normalized maximum amplitudes within the P phase time window. Light gray lines indicate the azimuth of the Utah FORGE stations available for the MT inversion. Dotted lines indicate nodal planes (±180 degrees) of the DC.

To analyze whether non-DC components can theoretically be resolved based on the available seismic network and the chosen frequency range, we design a resolution test using synthetic data^24^. We forward modeled waveforms of two representative microseismic events based on preliminary inversion results. The DCs of these events are a strike-slip mechanism and an oblique mechanism with a normal faulting component. We start with a pure DC solution and subsequently increase the ISO up to 35% while keeping the total seismic moment constant. Synthetic receivers are located on circles centered in the source location in azimuthal steps of 5$^\circ$. We test circle radii of 1, 2, and 4 km. Fig. 6 shows the results for 2 km which encircles the majority of Utah FORGE sensors used for the MT inversions (see also Supplementary Fig. S13-S16). To mimic the MT inversion setup, we cut short time windows of 0.6 s around the P and S phases and filtered the waveforms with a passband of 3–7 Hz. Then, we compare the obtained three-component waveforms modeled from the pure DC with the ones obtained using the increasing ISO at each synthetic receiver by computing the cross-correlation coefficient and the maximum amplitudes.

While these simple resolution tests do not account for noise conditions, site conditions, path effects, or unmodeled features of the velocity structure, they are helpful to understand the effect of ISOs on the radiation pattern and the resulting differences in the waveforms. If the forward modeled waveforms of the pure DC and the mixed mechanism with an ISO contribution do not show a distinct difference in amplitude and waveform shape in the given frequency range, it is impossible to invert for these ISO contributions under any circumstances^24^. At 2 km distance, waveform shapes and amplitudes of P phases differ significantly on radial and vertical components for a typical strike-slip event (Fig. 6 and Supplementary Fig. S13-S16). The results show a distinct azimuthal dependency of resolvability resulting from the strike-slip mechanism geometry and its symmetry, highlighting the importance of sufficient coverage of the focal sphere. At a distance of 4 km, waveform shape differences measured through cross-correlation are lower and mostly restricted to receivers close to the nodal planes. However, amplitude changes are still clearly observable. In the case of the oblique normal faulting event, the differences in waveform amplitudes and shape are even more clearly resolvable, especially for the P phase (Supplementary Fig. S15 and S16).

We find that the actual station distribution at Utah FORGE (grey lines in Fig. 6) sufficiently covers the critical azimuthal ranges with most prominent differences in waveform shape and maximum amplitudes. Under the given noise-free conditions, we estimate that it is theoretically possible to resolve ISOs of about $\sim$10% or more.

#### Summary of quality criteria for the interpretation of the ISO

From 229 candidate events, we obtained 189 MT solutions. The excluded 40 events did not pass our quality criteria, exhibiting, e.g., unstable solutions from the different bootstrap chains. Generally speaking, a small misfit of the single best solution for an MT does not necessarily mean that the true MT solution is found. Without considering the uncertainties and trade-offs among different MT parameters, non-uniqueness may bias the interpretations. Non-uniqueness may result, for example, from an insufficient station coverage, or a too narrow frequency band, when the recorded waveforms can be equally well fitted through different MTs, e.g., representing normal faulting or strike-slip events. This non-uniqueness can lead to an apparent heterogeneity in the microseismic mechanisms.

After the manual quality control, we report full MTs for 146 events and DC-only solutions for 43 events, in the case of unstable non-DCs. For the remaining full MTs, we subsequently require a bootstrap-based standard deviation of the ISO below 12% (Supplementary Fig. S4). The threshold was chosen to exclude solutions that are less well resolved, taking into account our synthetically modeled resolution of the ISO. Requiring a standard deviation of the isotropic component below 0.12 and carefully assessing the uncertainties and the resolution ability of our setup we assure that significant positive ISO of at least 15–20% are resolved, similar to thresholds from other studies^72^. The threshold ensures that there is no change of sign within one standard deviation, which would imply a flip from extension to compression. With this second criterion, we retain 109 events (57% of all MT solutions) for our interpretations regarding the ISO.

## Supplementary Information

Supplementary Information.

## Data Availability

Seismic waveform data of the UU network stations is publicly available from the fdsn server of EarthScope (https://service.iris.edu/fdsnws/). The nodal waveform stacks from the temporary data set are available as an assembled dataset at EarthScope (https://ds.iris.edu/ mda/24-037/). Data was processed and plotted using the open source software from pyrocko, Grond, matplotlib (https://matplotlib.org/matplotlib) and numpy (https://numpy.org/). The MT inversion results of this study (csv tables and html reports) are available via a supplementary zenodo repository (https://doi.org/10.5281/zenodo.15783808).
