# Supplementary material for: Isotropic components of microseismic moment tensors at Utah FORGE reveal a diversity of fluid pathway creation processes in EGS development
Source: Sci Rep. 2026 Mar 10;16:12916. doi: 10.1038/s41598-026-42493-0 (PMC13096226; doi:10.1038/s41598-026-42493-0)
Supplement: Supplementary file 1 — Supplementary Information. [file 41598_2026_42493_MOESM1_ESM.pdf]

Supplement

Isotropic components of microseismic moment tensors at Utah FORGE reveal a diversity of fluid pathway creation processes in EGS development

Peter Niemz*[1,4], Gesa Petersen[2], James Rutledge[3], Katherine Whidden[1], Kris Pankow[1]

[1] University of Utah, Seismograph Stations, Salt Lake City, UT, USA

[2] GFZ Helmholtz Centre for Geosciences, Potsdam, Germany

[3] Santa Fe Seismic LLC, 4 Entrada Empinada, Santa Fe, NM 87506, USA

[4] NORSAR, Gunnar Randers Vei 15, 2007 Kjeller, Norway

Corresponding author: P. Niemz: pniemz.seismology@gmail.com

Contents: This supplement contains additional information on the MT inversion results (Section S1; Fig. S1-S7) and on the dataset and methods (Section S2; Fig. S8-S16) that support the findings of the study.

S1. MT inversion results

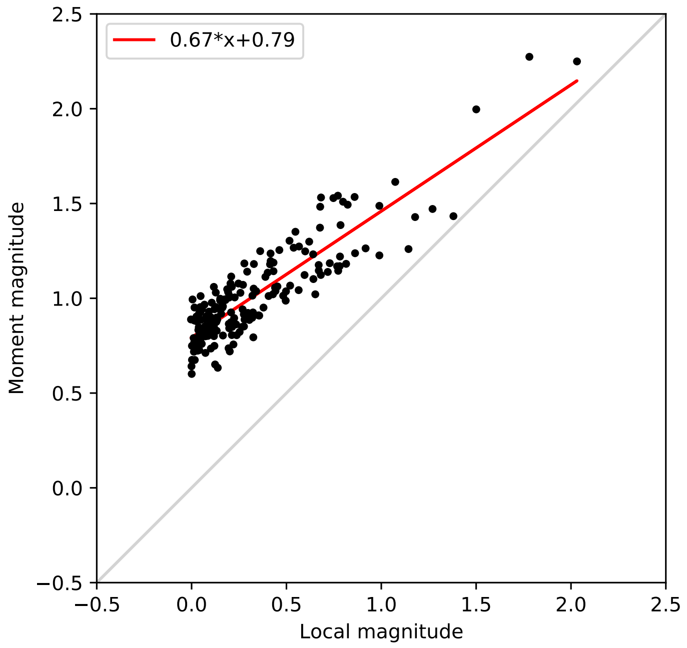

Figure S1: *Local magnitudes vs. moment magnitudes, the red line shows an L1-norm fitting to the points.*

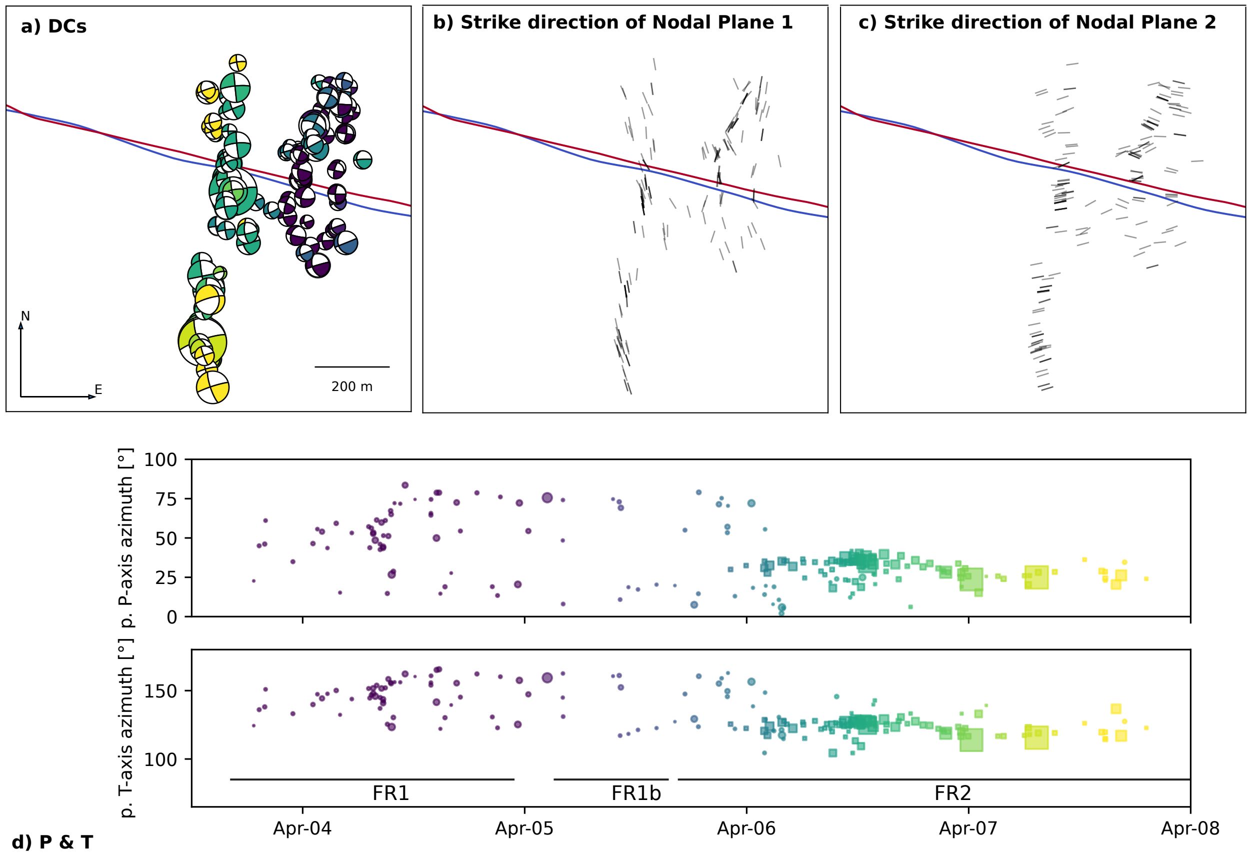

Figure S2: *Map view of MT inversion results, shown are (a) DC solutions, (b) strike directions of the nodal planes of all DCs which are north-south oriented, and (c) the nodal planes which are striking more in east-west direction. In (b) and (c) the nodal planes of all DCs from (a) are depicted. (d) Pressure and tension axes of the moment tensor solutions over time. FR1 and FR2 mark injection periods in the two features, dots and rectangles refer to events in these two distinct fracture zones. For simplicity and comparability, P-axis and T-axis from the western hemisphere are projected to the eastern side.*

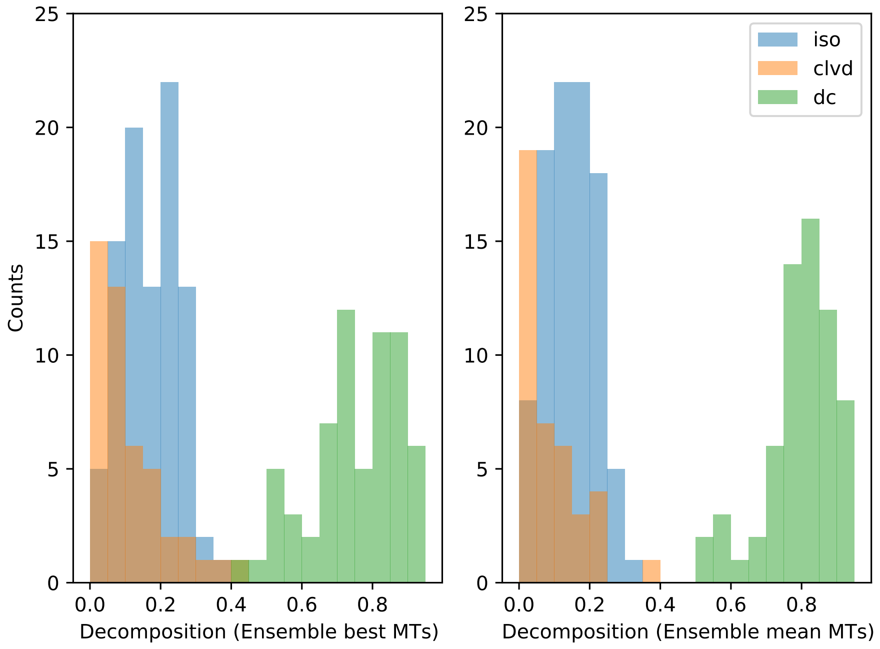

Figure S3: *(a) Decomposition of best MT solutions into DC, CLVD and isotropic component. (b) Mean decomposition of ensembles of solutions. Only MT solutions with a standard deviation below 0.12 for the isotropic component (see Fig. S4) are plotted.*

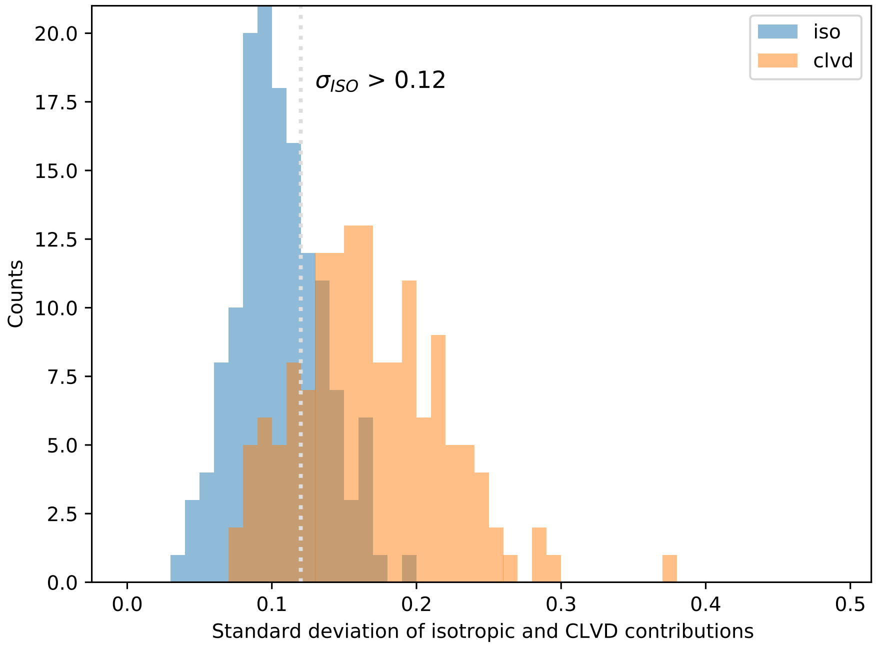

Figure S4: *Histograms showing standard deviations of ISO and CLVD components of ensemble means.*

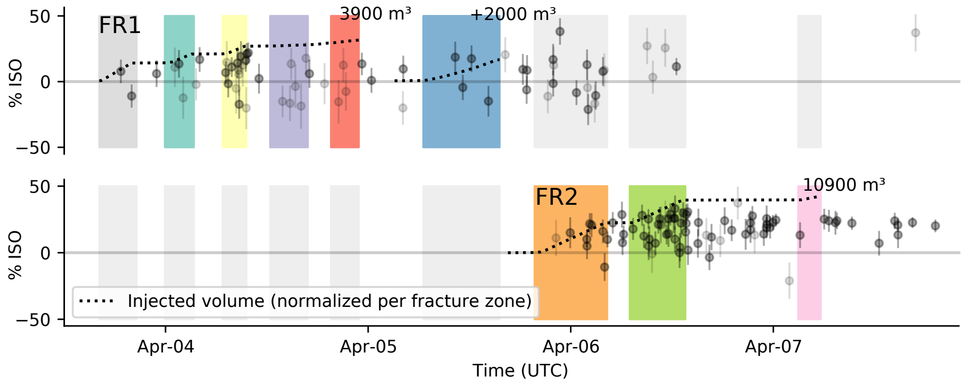

Figure S5: *Isotropic MT contribution and injected volume for fracture zone FR1 and FR2. Similar to Fig. 4 in the article, but including all MTs, irrespective of the ISO standard deviation. Error bars show the standard deviation of the isotropic contribution obtained from bootstrapping. The injected volume (dotted lines) is normalized per fracture zone. Stage S7 is normalized on its own as it formed a spatially distinct sub-feature within fracture zone FR1 (Niemz et al., 2025). Light grey points represent MTs with an ISO standard deviation below 12%. Background colors indicate the injection intervals as introduced in Fig. 1.*

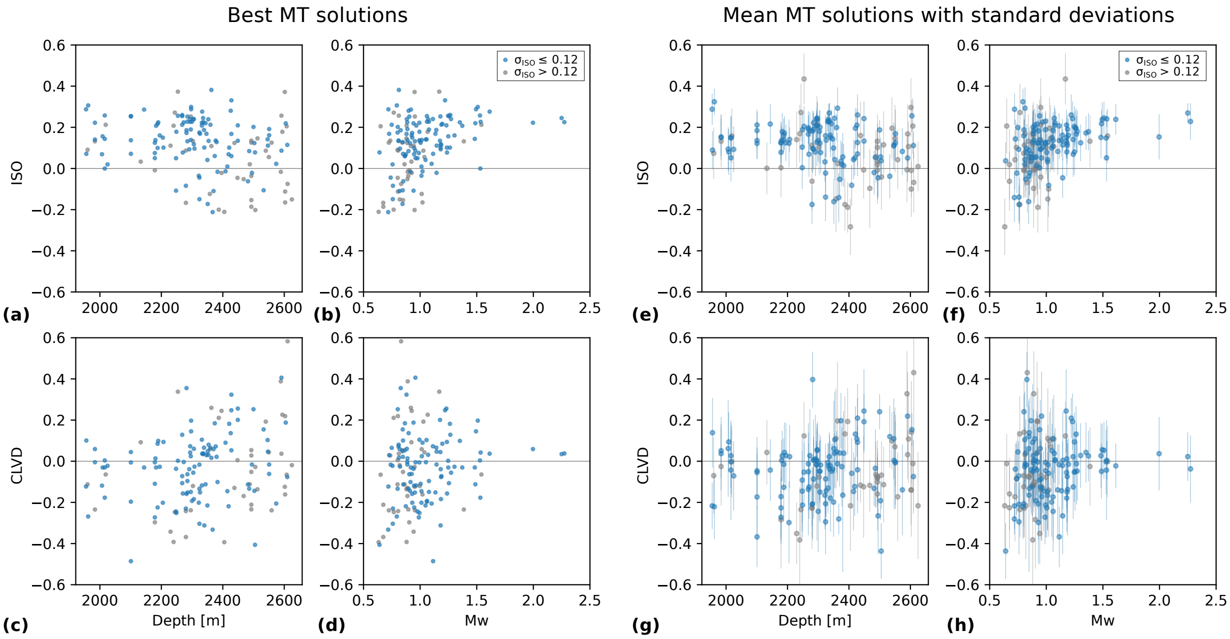

Figure S6: *Contribution of isotropic (top) and CLVD (bottom) component vs. depth and moment magnitude for best (left) and mean MT of the bootstrap ensembles with standard deviations (right); Events with a standard deviation of the isotropic component below 0.12 are plotted in blue, otherwise in gray. We do not observe any trend with depth or magnitude which would hint at a resolution problem.*

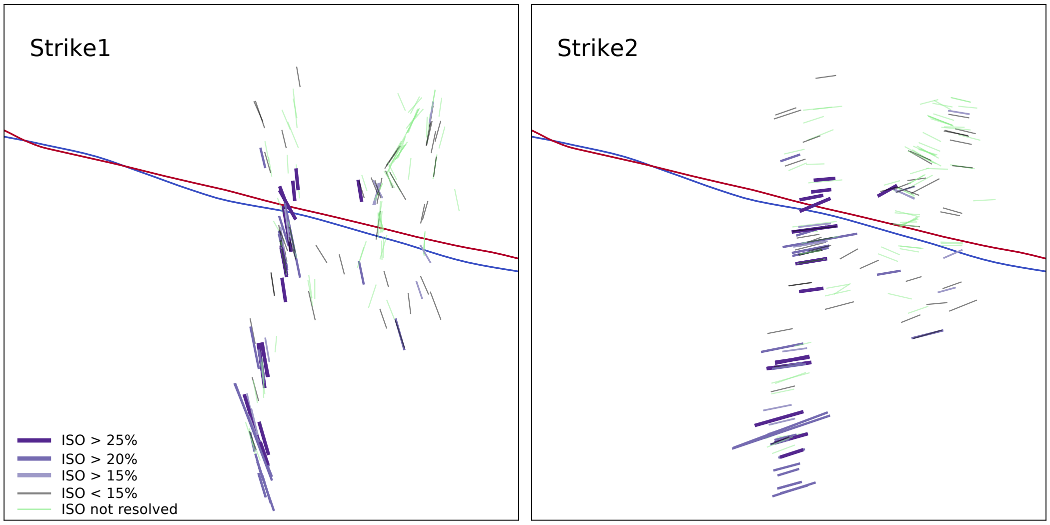

Figure S7: *Strike direction of nodal planes, color-coded by the contribution of the isotropic component. Fault plane length scaled by seismic moment assuming a constant stress drop of 1MPa (Madariaga, 1976).*

S2. Dataset & Methods

S2.1. Injection parameters

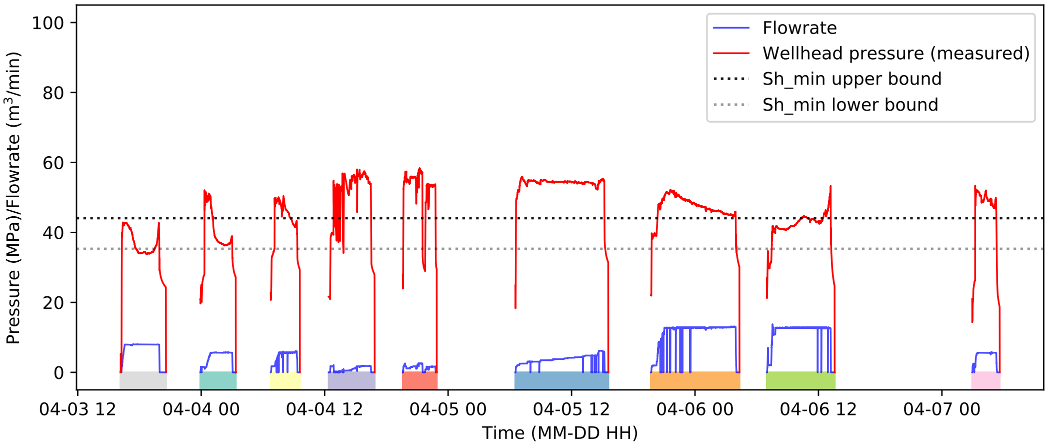

Figure S8: *Injection parameters of the 2024 stimulations at Utah FORGE. Xing et al. (2021) estimated local minimum horizontal stresses for Utah FORGE and changes with depth based on closure stress. Here we show upper and lower bounds for depths of 2200 m and 2600 m considering estimates from different methods (see Table 2 in Xing et al., 2021). We have no reliable estimates of downhole pressures but qualitatively speaking the downhole pressure is expected to be significantly larger due to the added hydrostatic pressure. Consequently, the downhole pressure at depth exceeds the lower and the upper bound of the minimum principal stress magnitude.*

S2.2. Quality control: Orientation of horizontal components

Misorientations of the horizontal components of seismometers are a common problem which can, if they are large, distort moment tensor inversions. In this study we rely on a nodal array that was set-up with hand-held compasses. Therefore we checked the sensor orientations using records of larger earthquakes at regional to teleseismic distances.

We apply the quality control toolbox AutoStatsQ (Petersen et al., 2019). The toolbox includes a test for sensor orientations based on the polarization of Rayleigh waves. Rayleigh waves are polarized with a phase shift of 90° between the vertical and radial components. In the test, waveforms of distant earthquakes are used. First, they are rotated into the Z,R,T coordinate system. Then, the horizontal traces are rotated in steps of 1°. For each step, the cross-correlation of the Hilbert-transformed vertical component (Z) and the rotated radial component (Rrot) is computed. If a sensor is correctly oriented the maximum cross-correlation value is reached without any additional rotation compared to the theoretical radial direction. We use a set of earthquakes from different azimuths and define the final misorientation based on the median of the results from all events. Using the median instead of the mean helps to avoid over-interpreting outlier results which can be biased by crustal-structures deviating the travel path.

Because of the short operation time of the nodal array (approx. 1 month), we cannot rely only on teleseismic, large ($M \geq 6.5$) earthquakes as suggested in Petersen et al. (2019) alone. We use eight earthquakes from a global catalog (Mw 5.8-7.4) at distances of 5000-13000 km and ten regional events from the USA (Mw 4.1-5.6) at 600-3000 km distance. For both groups of events we test different frequency filters and use those ones, which hold the best, most distinct results.

Regional catalog (USA)

- Tested frequency ranges: 0.02-0.06 Hz, 0.04-0.08 Hz, 0.06-0.10 Hz, 0.07-0.15 Hz
- Earthquake dataset: 10 Events (2024-04-05 -2024-05-01, Mw4.1 - 5.6, 600-3000 km)

Global catalog

- Tested frequency ranges: 0.01-0.04 Hz, 0.02-0.05 Hz, 0.02-0.06 Hz, 0.03-0.05 Hz, 0.04-0.06 Hz, 0.04-0.08 Hz

- Earthquake dataset: 8 Events (2024-03-27 -2024-04-29, Mw5.8- 7.4, 5000-13000 km)

The resulting median misorientations of all sensors are well below 10° (Fig. S9). Therefore we consider the sensors sufficiently well oriented for further analysis.

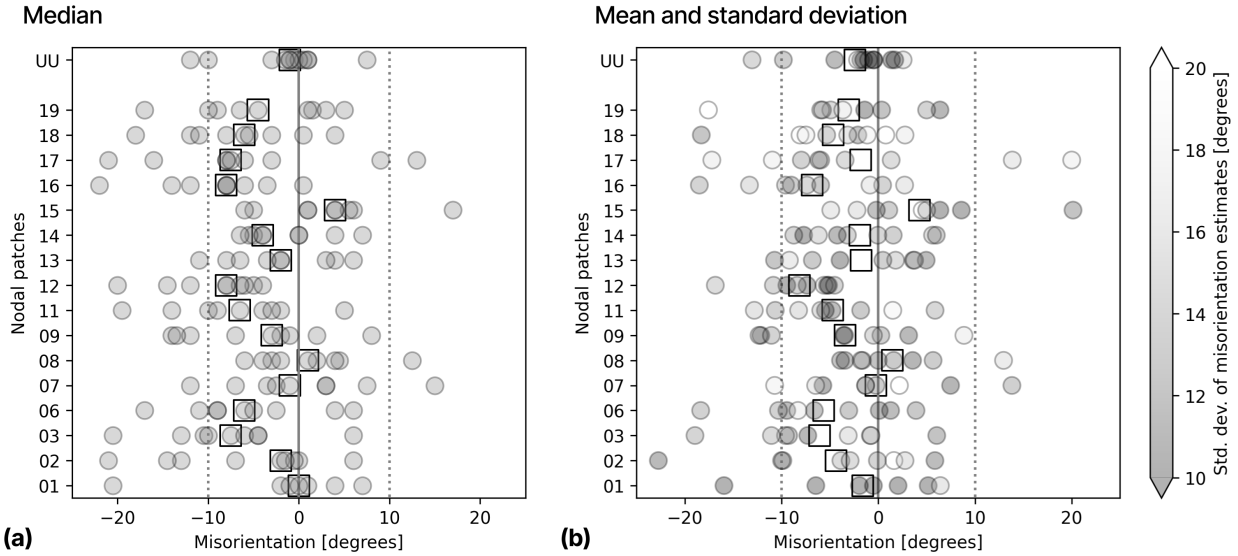

Figure S9: *Orientation test of horizontal components: (a) Median misorientation and (b) mean misorientation of the geophones sorted by patch based on the combined results of teleseismic and regional catalogs. The squares mark the median/mean misorientation in each patch. Misorientation below 10 degrees (dotted lines) are considered insignificant for our analysis. For reference, orientations of UU network stations at Utah FORGE are shown on a single line at the top. In (b) dots are colored by standard deviation.*

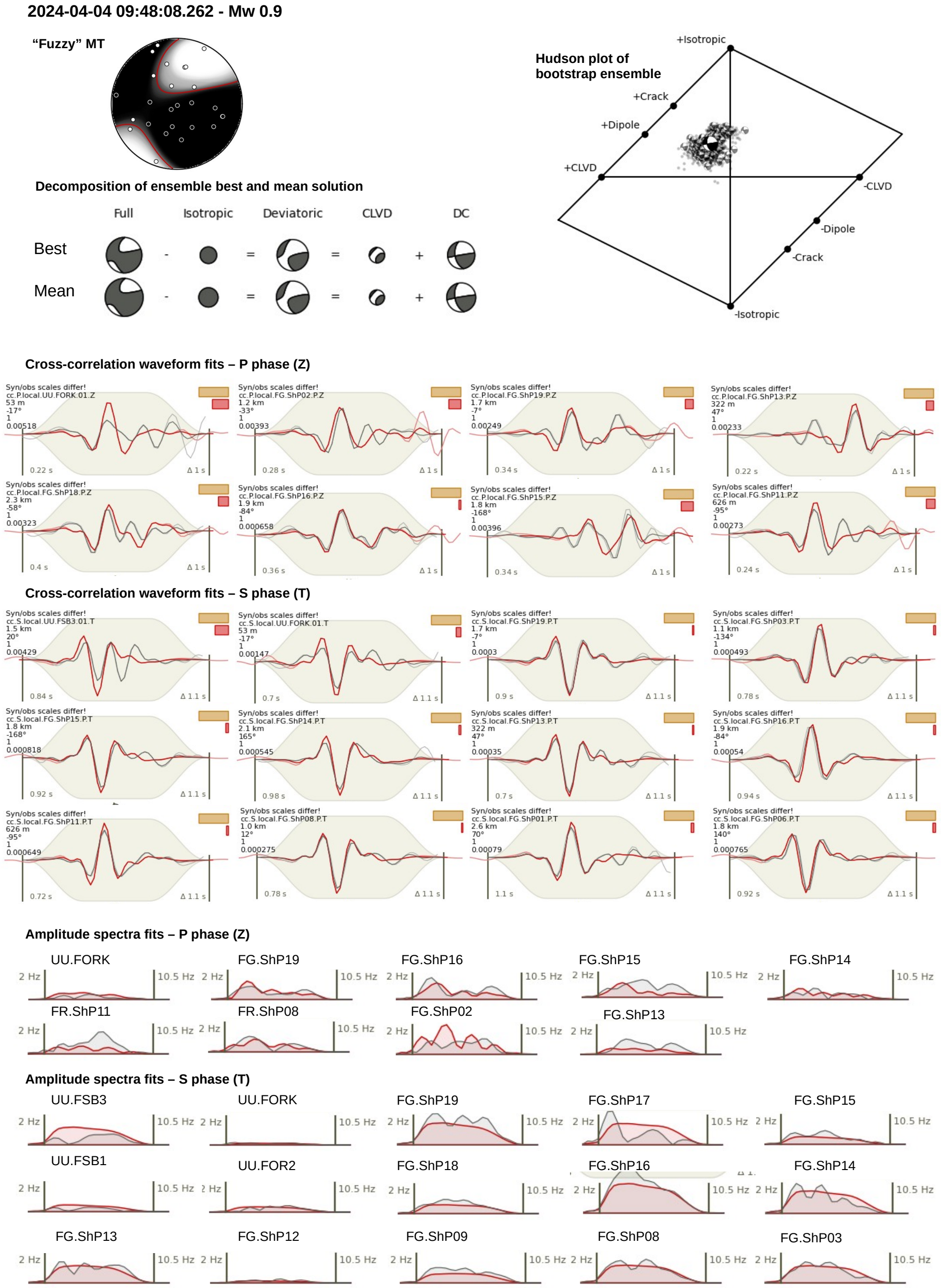

Figure S10: *Example MT inversion with a stable result. The "fuzzy MT" (top left) is an overlay of the retrieved P-wave radiation pattern of the ten best solutions of each of the 101 bootstrap chains. The comparison of the decompositions of the best and mean MT of the bootstrap ensemble below provides graphical information on the stability of the solution. The Hudson diagram (top right) is used to visualize the scatter of non-DC components within the ensemble of bootstrap solutions(101 chains x 10 best solutions). The bottom figures show example waveform fits for cross-correlation-based fitting of P phase and S phases on vertical and transversal components, respectively, and spectral fits of P and S phases on vertical and transversal components, respectively. Red colors indicate synthetics, gray the observations. Shown are misfits of the best model of the global chain. The waveforms shown in cross-correlation fitting examples are normalized. Labels next to the plots indicate epicentral station-event distance, azimuth, weighting, and contribution to the misfit. Labels left and right of the spectra indicate the minimum and maximum frequency, resulting from a taper of 1.5 times the chosen frequency to avoid abrupt cut-offs.*

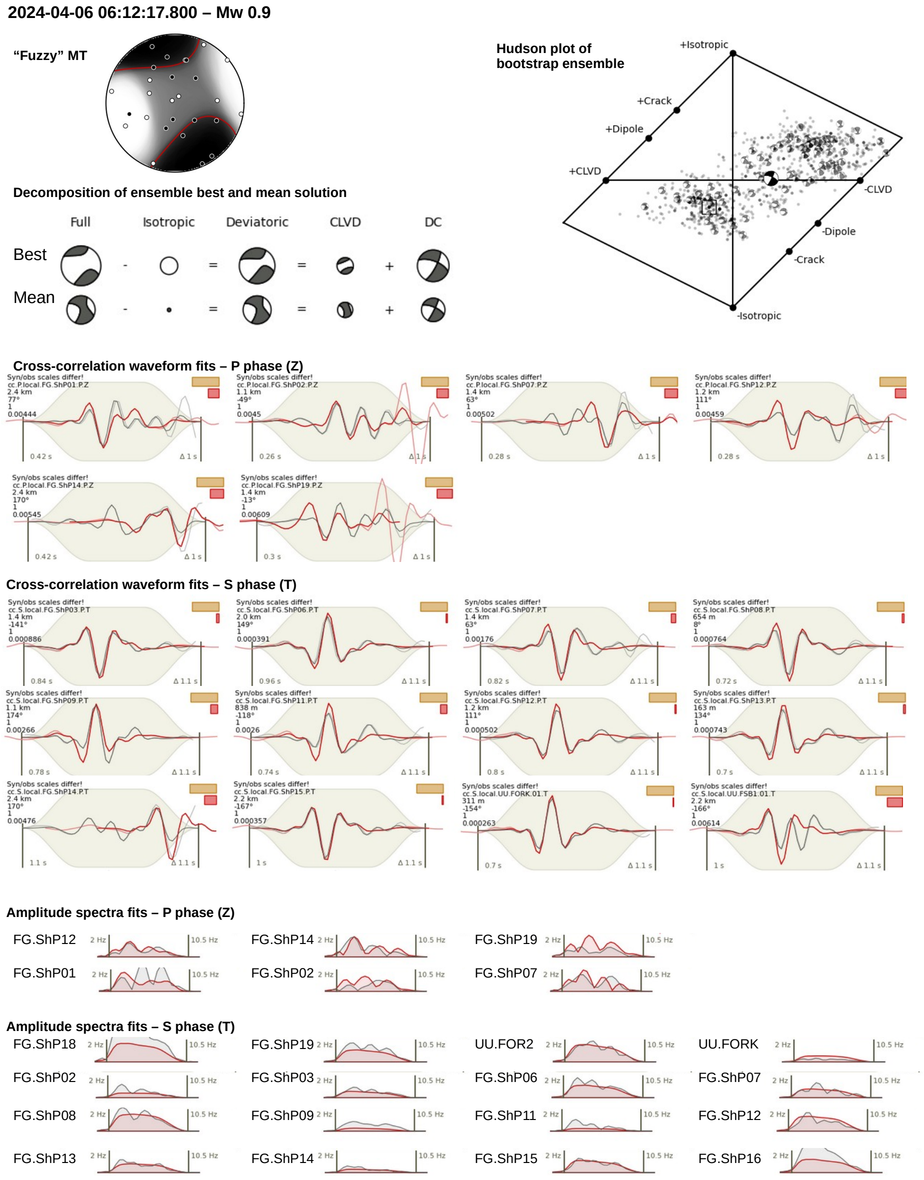

Figure S11: *Example MT inversion result that was* ***excluded*** *because it was not considered sufficiently stable for interpretations. The "fuzzy beachball" (top left) is an overlay of the retrieved P-wave radiation pattern of the ten best solutions of each of the 101 bootstrap chains. The comparison of the decompositions of the best and mean MT of the bootstrap ensemble below provides graphical information on the stability of the solution. The Hudson diagram (top right) is used to visualize the scatter of non-DC components within the ensemble of bootstrap solutions. The bottom figures show example waveform fits for cross-correlation-based fitting of P phase and S phases on vertical and transversal components, respectively, and spectral fits of P and S phases on vertical and transversal components, respectively. See previous figure for more information.*

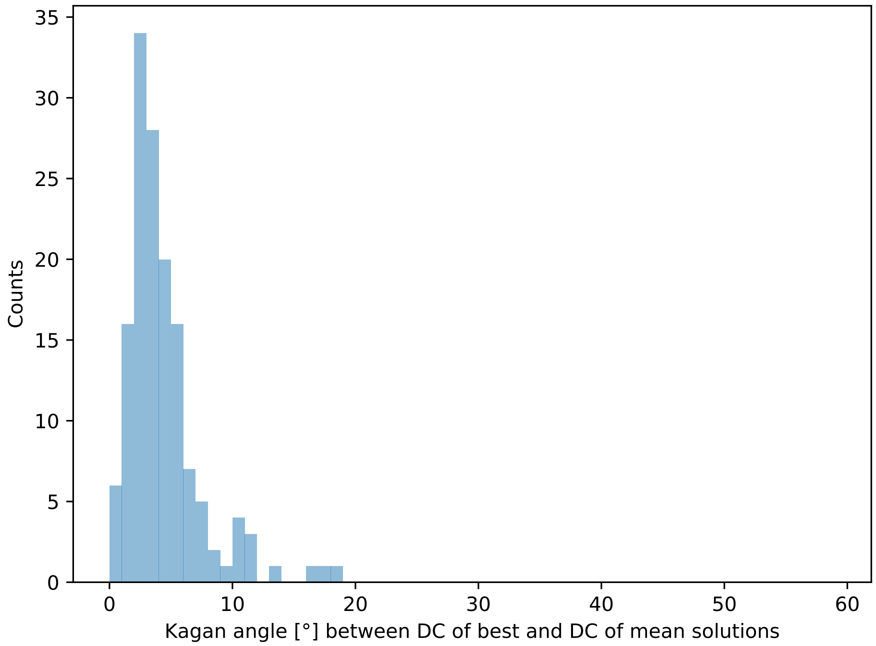

Figure S12: *Kagan angle between best solution and mean solution of each event showing the great stability of the double-couple components of our MT inversions.*

S2.3. Synthetic waveform-based resolution tests

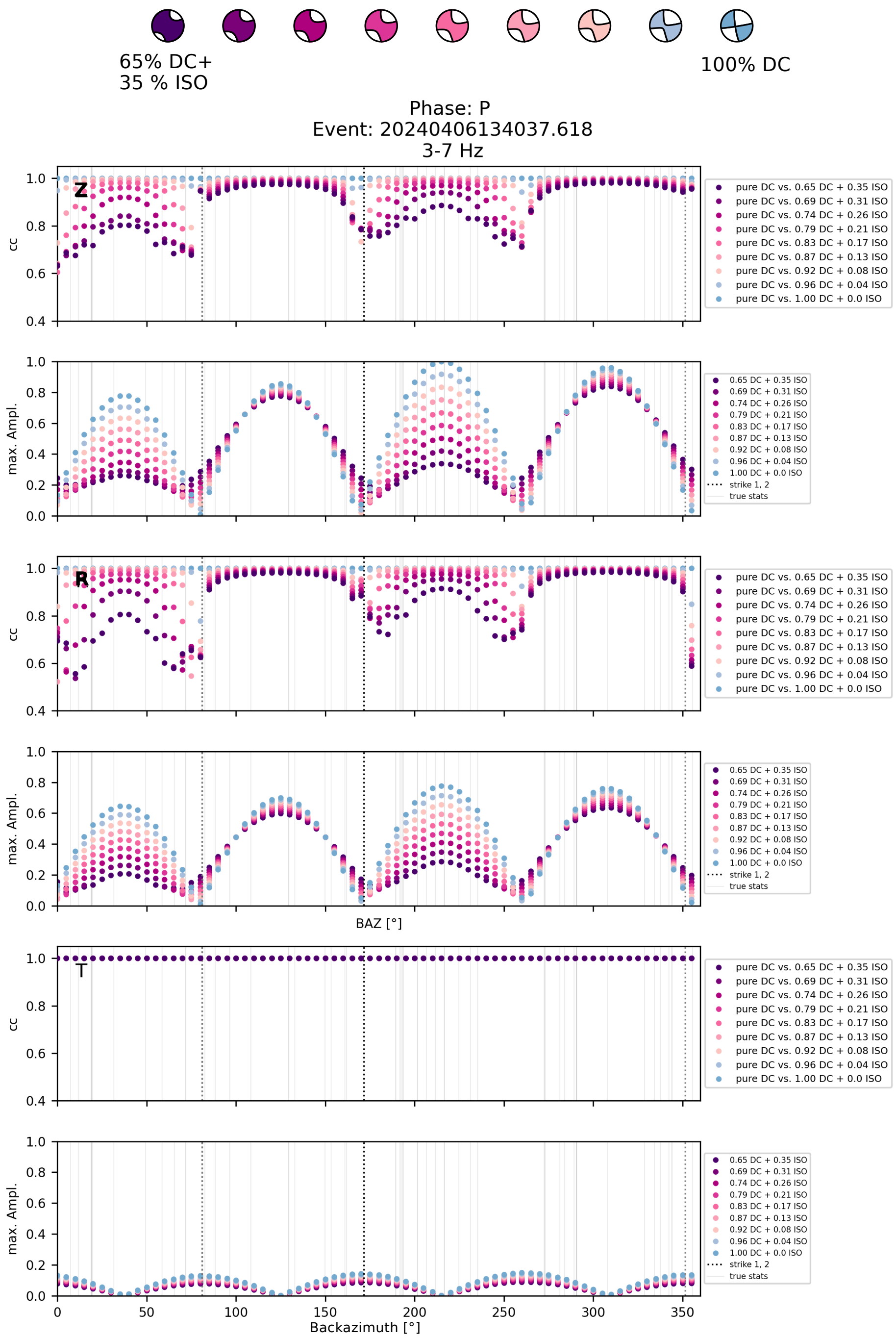

Figure S13: *P-phase synthetic-waveform-based resolution test for isotropic components using a typical strike-slip source with a variable isotropic component of 0% to 35%. Synthetic receivers are located on a circle in 2 km distance from the event, every 5°. From top to bottom: vertical (Z), radial (R) and transversal (T) component. For each component the upper panel shows the comparison of waveforms through cross-correlation of a pure double-couple source (blue) and mixed-mechanisms generated from the same double-couple and a positive isotropic component (see colored focal mechanisms on top). The lower panels of each component show the normalized maximum amplitudes of all source types within the P phase time window. Light gray lines indicate the azimuth of the Utah FORGE stations used in the MT inversion. Dotted lines indicate nodal planes (+- 180 degrees) of the DC.*

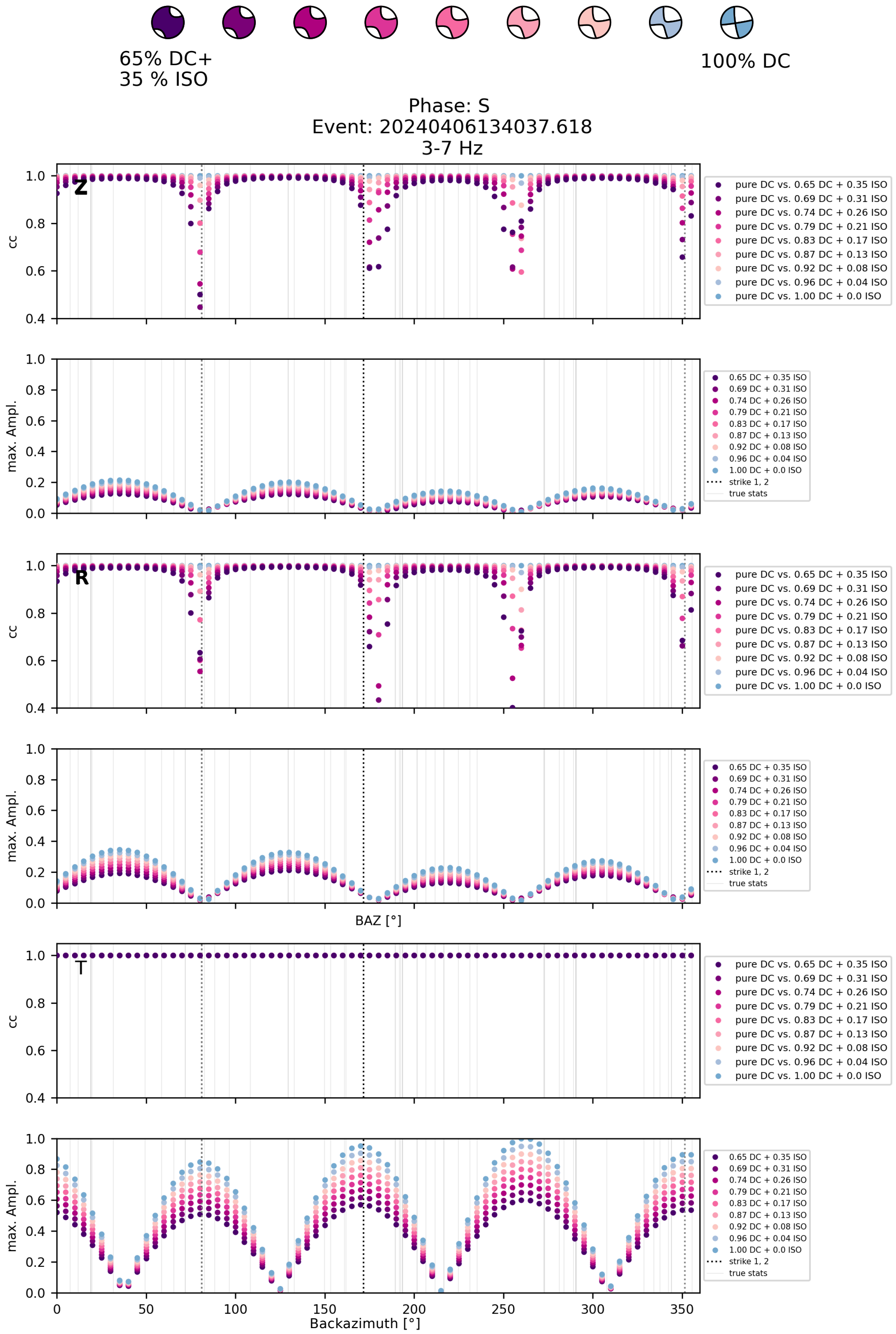

Figure S14: *Same as previous figure, but time windows containing S-phases.*

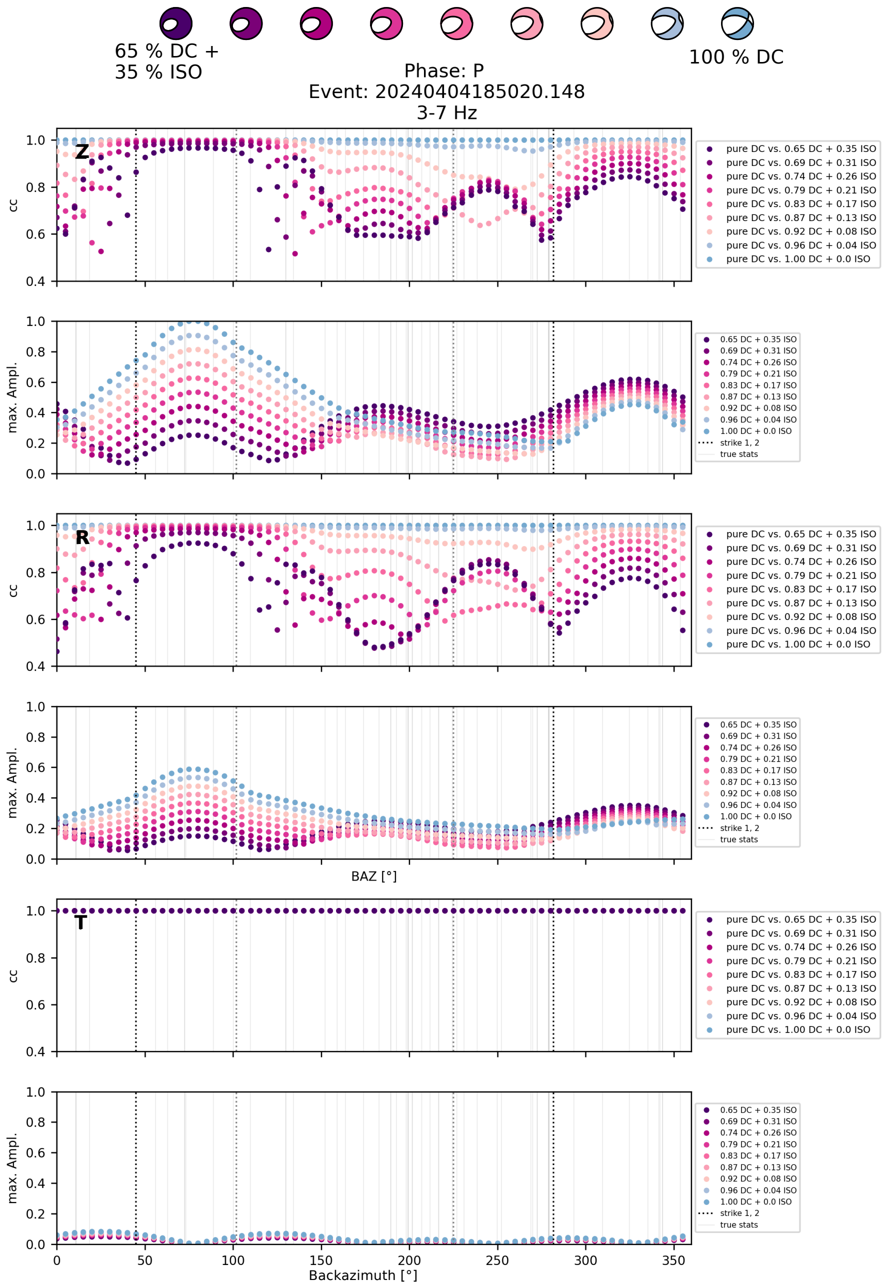

Figure S15: *Same as previous figures, shown is the test result for a normal faulting event, P phase time windows.*

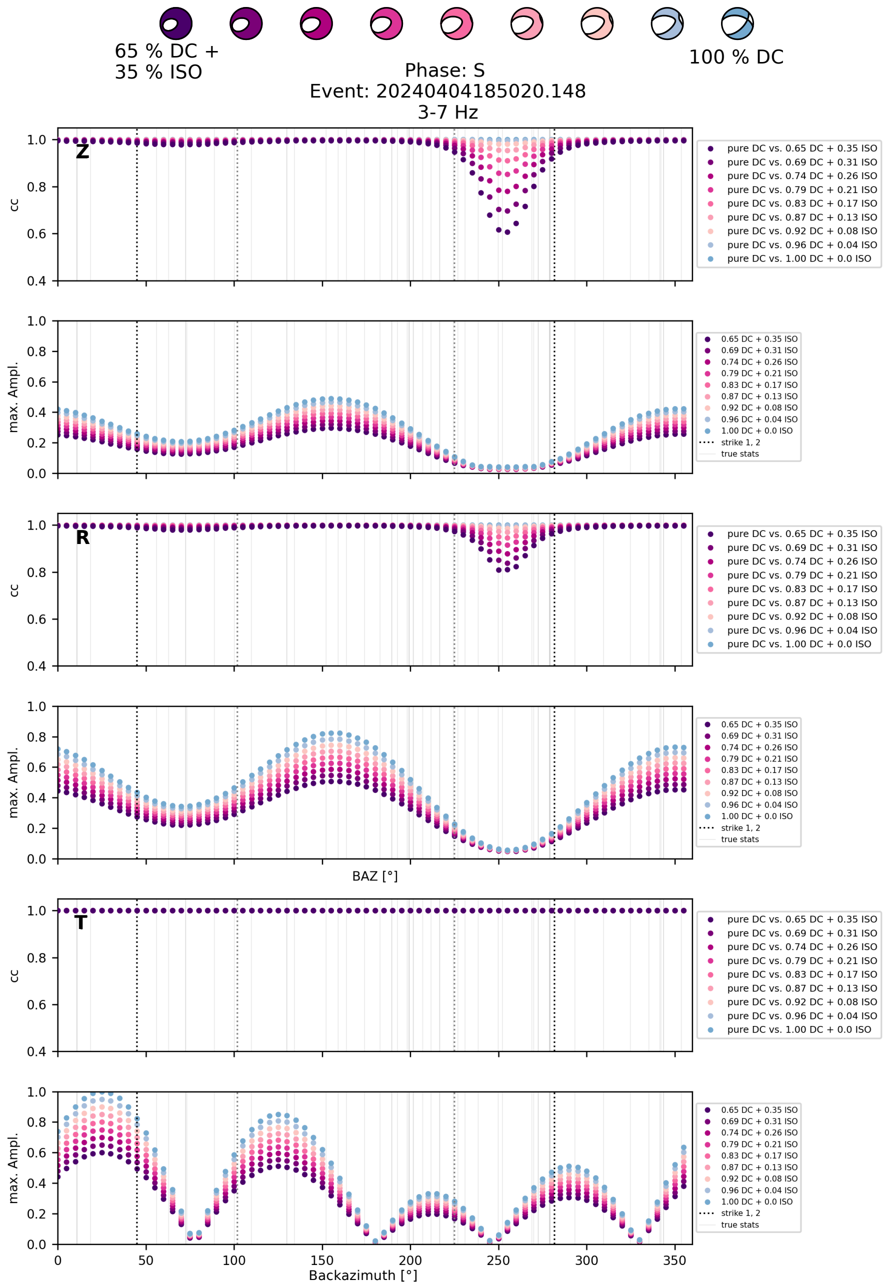

Figure S16: *Same as previous figures, shown is the test result for a normal faulting event, S phase time windows.*

References

R. Madariaga. Dynamics of an expanding circular fault. *Bulletin of the Seismological Society of America*, 66 (3):639–666, 06 1976. ISSN 0037-1106. doi:10.1785/BSSA0660030639. URL `https://doi.org/10.1785/BSSA0660030639`.

P. Niemz, K. Pankow, M. P. Isken, K. Whidden, J. McLennan, and J. Moore. Mapping fracture zones with nodal geophone patches: Insights from induced microseismicity during the 2024 stimulations at utah forge. *Seismological Research Letters, doi: https://doi. org/10.1785/0220240300*, 96(3):1603–1618, 2025.

G. M. Petersen, S. Cesca, M. Kriegerowski, and the AlpArray Working Group. Automated Quality Control for Large Seismic Networks: Implementation and Application to the AlpArray Seismic Network. *Seismological Research Letters*, 90(3):1177–1190, 2019. doi:10.1785/0220180342.

P. Xing, D. Winkler, L. Swearingen, J. Moore, and J. McLennan. In-Situ Stresses and Permeability Measurements from Testings in Injection Well 16A(78)-32 at Utah FORGE Site. *GRC Transactions*, 45, 2021.
